# Differences in the prevalence and patterns of dementia risk factors across 14 countries and regions: a harmonised cross-national analysis

**DOI:** 10.1016/j.lanhl.2026.100867

**Published:** 2026-07-12

**Authors:** Emma Nichols, Zachary J Kunicki, Michael Markot, Drystan Phillips, Jenny Wilkens, Alden L Gross, Jinkook Lee

**Affiliations:** **Center for Economic and Social Research, University of Southern California, Los Angeles, CA, USA** (E Nichols PhD, M Markot MPH, D Phillips MA, J Wilkens MPH, Prof J Lee PhD)**; Leonard Davis School of Gerontology, University of Southern California, Los Angeles, CA, USA** (E Nichols)**; Department of Economics, University of Southern California, Los Angeles, CA, USA** (Prof J Lee)**; Department of Psychiatry and Human Behavior, Warren Alpert Medical School of Brown University, Providence, RI, USA** (Z J Kunicki PhD)**; Johns Hopkins Center on Aging and Health** (Prof A L Gross PhD) **and Department of Epidemiology** (Prof A L Gross)**, Johns Hopkins Bloomberg School of Public Health, Baltimore, MD, USA**

## Abstract

**Background:**

The design of evidence-based interventions to reduce the burden of dementia requires knowledge of the prevalence and patterns of modifiable risk factors. However, most existing evidence comes from high-income countries (HICs). Hence, we aimed to quantify differences in the prevalence and patterns of dementia risk factors across diverse contexts.

**Methods:**

We conducted a comparative cross-sectional study using harmonised data from 14 countries and regions (Ireland, the USA, England, Northern Ireland, Eastern Europe, Western Europe, Northern Europe, Southern Europe, South Korea, Mexico, China, Malaysia, Brazil, and India), including HICs and low-income and middle-income countries (LMICs). We included individuals aged 50 years or older from 11 nationally representative ageing studies, using data from the most recent available study waves with refresher samples collected between 2009 and 2023. Respondents were excluded from specific analyses if data on the risk factors of interest were missing. We estimated the prevalence of 12 established binary dementia risk factors (low education, hearing loss, high LDL cholesterol, depression, physical inactivity, diabetes, smoking, hypertension, obesity, excessive alcohol consumption, social isolation, and vision loss) using descriptive statistics and examined patterns by age group (70 years and older and 50–69 years), gender, and education using Poisson models with robust variance estimation. We also compared the rank order of the prevalence of risk factors across countries and assessed risk factor co-occurrence and clustering.

**Findings:**

Data on 214 251 respondents were included in the study. We observed some variation in the prevalence and patterns (by age, gender, and education) of risk factors between HICs and LMICs. For example, low education had higher prevalence in many LMICs (85·6% [95% CI 84·8–86·5] in China *vs* 12·0% [95% CI 11·3–12·7] in the US), whereas obesity was more prevalent in HICs than in LMICs (44·9% [95% CI 43·3–46·5] in the US *vs* 13·3% [95% CI 12·9–13·7] in India). Risk factor distributions differed by age group, gender, and education, although patterns were not consistent across all settings. Risk factors commonly co-occurred across settings, with more than 50% of individuals having at least two risk factors across all countries and regions. Moreover, broadly similar clusters of risk factors—related to cardiovascular diseases, risky behaviours, and social or sensory factors—were observed across settings.

**Interpretation:**

Differences in the prevalence and patterns of dementia risk factors highlight the need to tailor prevention strategies to specific contexts. However, findings also reveal consistent patterns in risk factor co-occurrence and clustering, which could guide the design of multidomain interventions and policy approaches to reduce dementia risk across settings. Overall, these findings support the use of both context-specific and shared approaches to reduce the burden of dementia.

**Funding:**

National Institutes of Health.

## Introduction

Dementia prevalence is expected to triple by 2050, largely because of population ageing.^1^ However, there is increasing recognition that a large proportion of dementia burden is attributable to modifiable life course risk factors.^2^ According to the 2024 update of the *Lancet* Commission report, 45% of dementia burden is attributable to 14 modifiable risk factors—namely, low education, hearing loss, high LDL cholesterol, depression, traumatic brain injury, physical inactivity, diabetes, smoking, hypertension, obesity, excessive alcohol use, social isolation, air pollution, and vision loss.^3^ Understanding the prevalence and patterns of these risk factors is key for designing effective evidence-based interventions to reduce dementia burden.

Most evidence underlying the recommendations in the *Lancet* Commission report comes from high-income countries (HICs),^3^ but the need for data from low-income and middle-income countries (LMICs) is increasingly acknowledged, given potential differences across settings and contexts (ie, countries or regions with differing demographic, socioeconomic, and health system characteristics).^4,5^ For example, by integrating evidence on differences in the prevalence of risk factors across contexts, the proportion of dementia that was potentially modifiable was estimated to range from 40% in China to 56% in Latin America.^6^ More detailed country-specific analyses further evaluate the effect of common assumptions (eg, that the effects of risk factors do not differ across contexts) on estimates of modifiable dementia burden, with one study suggesting that up to 70% of dementia might be modifiable in India.^7^

Existing research on dementia risk factors in diverse settings has largely focused on population attributable fractions (PAFs) in specific countries (eg, Chile^8^ and Brazil^9^) without explicitly examining how risk factors coexist or how their prevalence and patterns vary. However, risk factors commonly co-occur and cluster together,^10,11^ and this co-occurrence contributes to dementia risk.^12^ Although this evidence on co-occurring risk factors has motivated the development of multidomain intervention trials, such as the Finnish Geriatric Intervention Study to Prevent Cognitive Impairment and Disability (FINGER) and worldwide FINGER trials,^13,14^ comparative data on risk factor prevalence and patterns across contexts to guide the development or scale-up of policies or interventions are scarce.

This study aimed to fill this evidence gap by quantifying differences in the prevalence and patterns of 12 established dementia risk factors across 14 countries and regions in diverse global contexts to inform cross-country comparisons. To maximise comparability between contexts, we leveraged nationally representative harmonised data collected between 2009 and 2023. Findings can inform how policies and interventions targeting dementia risk factors should be tailored to varying country contexts.

## Methods

### Study design

In this comparative cross-sectional study, we used data from 11 nationally representative studies on ageing from the Health and Retirement Study International Network of Studies (HRS INS), a network of global aging studies designed to facilitate cross-national comparisons. To maximise comparability across datasets, we used harmonised HRS INS data from the Gateway to Global Ageing Data.^15^ This study adhered to the STROBE guidelines.

Data from The Irish Longitudinal Study on Ageing (TILDA), the Health and Retirement Study (HRS), the English Longitudinal Study of Ageing (ELSA), the Northern Ireland Cohort for the Longitudinal Study of Ageing (NICOLA), the Survey of Health, Ageing and Retirement in Europe (SHARE), the Korean Longitudinal Study of Ageing (KLoSA), the Mexican Health and Aging Study (MHAS), the China Health and Retirement Longitudinal Study (CHARLS), the Malaysia Ageing and Retirement Survey (MARS), the Brazilian Longitudinal Study of Ageing (ELSI), and the Longitudinal Ageing Study in India (LASI) were included (appendix p 3). Study-specific details are available in the appendix (pp 4-5). To account for cultural and contextual differences across Europe, we divided SHARE into four European regions (north [hereafter SHARE N], south [SHARE S], east [SHARE E], and west [SHARE W]) based on UN Statistics Division classification.^16^ Thus, we analysed 14 countries or regions—namely, Ireland, the USA, England, Northern Ireland, Eastern Europe, Western Europe, Northern Europe, Southern Europe, South Korea, Mexico, China, Malaysia, Brazil, and India. We excluded respondents younger than 50 years to standardise the age inclusion criteria.

We used data from the most recent study wave with a refresher sample (hereafter referred to as the primary wave) for most source datasets to maximise policy relevance while ensuring representativeness. The primary study waves were conducted between 2009 and 2023 (appendix p 4). For some studies, data from older waves were used for the analyses of specific risk factors (details in appendix pp 6-16). All studies used multistage probability sampling to ensure population representativeness. Each study received approval from local ethics committees, and informed consent was obtained from all respondents. No ethics approval was required for this study.

### Risk factors and covariates

We included 12 of 14 dementia risk factors identified in the *Lancet* Commission report and commonly available in the HRS INS. These risk factors were low education, hearing loss, high LDL cholesterol, depression, physical inactivity, diabetes, smoking, hypertension, obesity, excessive alcohol consumption, social isolation, and vision loss. Traumatic brain injury and air pollution were excluded because the data were unavailable.

Respondents self-reported details on their education, smoking status, physical activity levels, and alcohol consumption. Education categories were standardised based on International Standard Classification of Education (ISCED) guidelines.^17^ For this study, we considered education below the level of upper secondary education as low education. For primary analyses, we defined smoking as current or former smoking. We used established equations to estimate the Leisure Score Index (LSI) based on vigorous, moderate, and light activity and defined low physical activity as activity with a LSI of less than 14 (appendix pp 9-10).^18^ We defined excessive alcohol use as the consumption of 15 or more drinks per week for men and the consumption of eight or more drinks per week for women (appendix pp 13-15).^19^ High cholesterol, diabetes, and hypertension data were based on self-reported physician diagnosis. Hearing and vision loss were self-reported using survey questions with binary or Likert-scale response options (appendix pp 6-7, 16-17); ratings of fair or poor were classified as hearing or vision loss in all cases except for KLoSA and CHARLS. In KLoSA and CHARLS, differences in the distribution of responses suggested that respondents interpreted fair as adequate, rather than poor, and this was classified accordingly; the term fair when directly translated to Korean or Mandarin is commonly interpreted as adequate or reasonable, aligning with our descriptive observations and supporting our dichotomisation strategy.

Symptoms of depression were assessed using the Center for Epidemiologic Studies Depression Scale in most studies, with context-specific cutoffs based on existing literature.^20^ In SHARE, the EURO-D and its corresponding cutpoint were used instead (appendix pp 7-9).^21^ Because of the differences in the questions and response scales used to assess social isolation across studies, we used a simplified definition for social isolation based on living arrangements and classified individuals who live alone as socially isolated. Obesity was defined using two cutpoints. Based on the evidence indicating the need for alternative BMI cutpoints in Asian populations,^22^ we used a threshold of 27·5 for CHARLS, KLoSA, MARS, and LASI data and a threshold of 30 for other datasets. BMI was based on measured height and weight when these parameters were available, owing to the low risk of bias, and on self-reported height and weight in KLoSA. In MHAS, measured BMI from a previous wave was used in analyses examining individual risk factors, whereas BMI from self-reported data in the primary wave was used in analyses examining the joint distribution of risk factors. Additional details are available in the appendix (pp 6-16).

We used self-reported age and sex (hereafter gender) as covariates.

### Statistical analysis

We used descriptive statistics to examine the prevalence of each binary risk factor. We also calculated the prevalence and means across risk factor definitions in sensitivity analyses. We used sorted prevalence estimates to calculate ranks and visualised the rank order of risk factor prevalences using a heatmap. To examine patterns among risk factors by age, gender, and education, we used study-specific and risk-specific Poisson models with Horvitz–Thompson-type robust standard errors (SE) to estimate risk ratios. Models were mutually adjusted for all three variables. To evaluate whether education patterns differed by gender, we ran follow-up models with interaction terms between education and gender.

In analyses of the joint distribution of risk factors, we excluded risk factors that were not available in the primary wave. We first calculated the weighted proportion of individuals with more than two, three, or four risk factors. Given differences in the number of risk factors available by study, we also estimated the mean number of co-occurring risk factors as a proportion of available risk factors for each study. Additionally, we identified risk factor clusters. We used parallel analysis to select the number of risk factor clusters in each study. Based on principal component analysis with varimax rotation, we assigned each risk factor to the component with the highest loading and named the clusters based on the included risk factors. We used a heatmap to visualise findings and compare patterns of clustering across countries and regions.

We conducted six sensitivity analyses intended to evaluate the effect of decisions related to the measurement of risk factors on prevalence estimates. First, we examined the prevalence of secondary and tertiary education and of low education. Second, we reported the prevalence of three smoking categories (never, former smoker, and current smoker). Third, we evaluated an alternative strategy to convert categorical data on physical activity to the LSI (appendix pp 9-10), while also reporting vigorous, moderate, and light activity separately. Fourth, because LASI did not include questions on the quantity of alcohol consumption comparable to those in other studies, we additionally evaluated a binary indicator for binge alcohol consumption across all surveys (appendix pp 15). We also evaluated potential self-report biases in cardiovascular disease risk factors by comparing objectively measured hypertension (or self-reported blood pressure medication use) with self-reported physician diagnosis of hypertension when data were available. To test if this bias was related to development status, we estimated the Pearson correlation between log-transformed gross domestic product (GDP) per capita and the difference between objective and self-reported hypertension prevalence. Finally, we considered versions of hearing and vision loss measures in KLoSA and CHARLS without applying language translation adjustments.

To identify patterns related to differences in economic contexts, we ordered studies by country GDP per capita for all results. A significance threshold of α=0·05 was used where statistical testing was applied; however, formal hypothesis testing was not conducted systematically across all analyses. All analyses used survey weights and accounted for survey design features when available (appendix p 17). For all analyses, we excluded respondents with missing data on risk factors of interest; principal component analysis used correlation matrices constructed from non-missing data on pairwise comparisons. Missing data were generally minimal, except for health variables in NICOLA and physical measures in HRS and MHAS (appendix pp 18-29). Because high levels of missingness in these variables were attributable to study design (these measures were not collected for the full sample), this missingness is less likely to be informative or cause bias in the results. All analyses were conducted using R version 4.5.1.

### Role of the funding source

The funders had no role in the study design, data collection, data analysis, data interpretation, or writing of the report.

## Results

We included 214251 respondents from 14 countries and regions in the primary wave with non-zero survey weights and available data on at least one risk factor. Prevalence varied substantially across settings for most dementia risk factors (table). The prevalence of low formal education showed large variability, ranging from 85·6% (95% CI 84·8–86·5) in CHARLS (China) to 12·0% (95% CI 11·3–12·7) in HRS (the USA). Although low education was most prevalent in LMICs, other risk factors showed different patterns. For example, the prevalence of obesity was the highest in HRS (the USA; 44·9%, 95% CI 43·3–46·5), followed by MHAS (Mexico; 35·7%, 95% CI 31·5–39·9), and the lowest in CHARLS (China; 14·5%, 95% CI 13·7–15·3), LASI (India; 13·3%, 95% CI 12·9–13·7), and KLoSA (South Korea; 6·2%, 95% CI 5·6–6·8). Cardiovascular disease risk factors were more prevalent in HICs than in LMICs. The prevalence of cardiovascular disease risk factors, such as hypertension, was also higher in the two Latin American countries than in other countries with similar socioeconomic status. For example, the prevalence of hypertension was 53·1% (95% CI 51·5–54·7) in MHAS (Mexico) and 49·0% (95% CI 47·7–50·3) in ELSI (Brazil), compared with 40·2% (95% CI 39·1–41·2) in CHARLS (China) and 43·4% (95% CI 41·8–44·9) in MARS (Malaysia).

**Figure 1:**
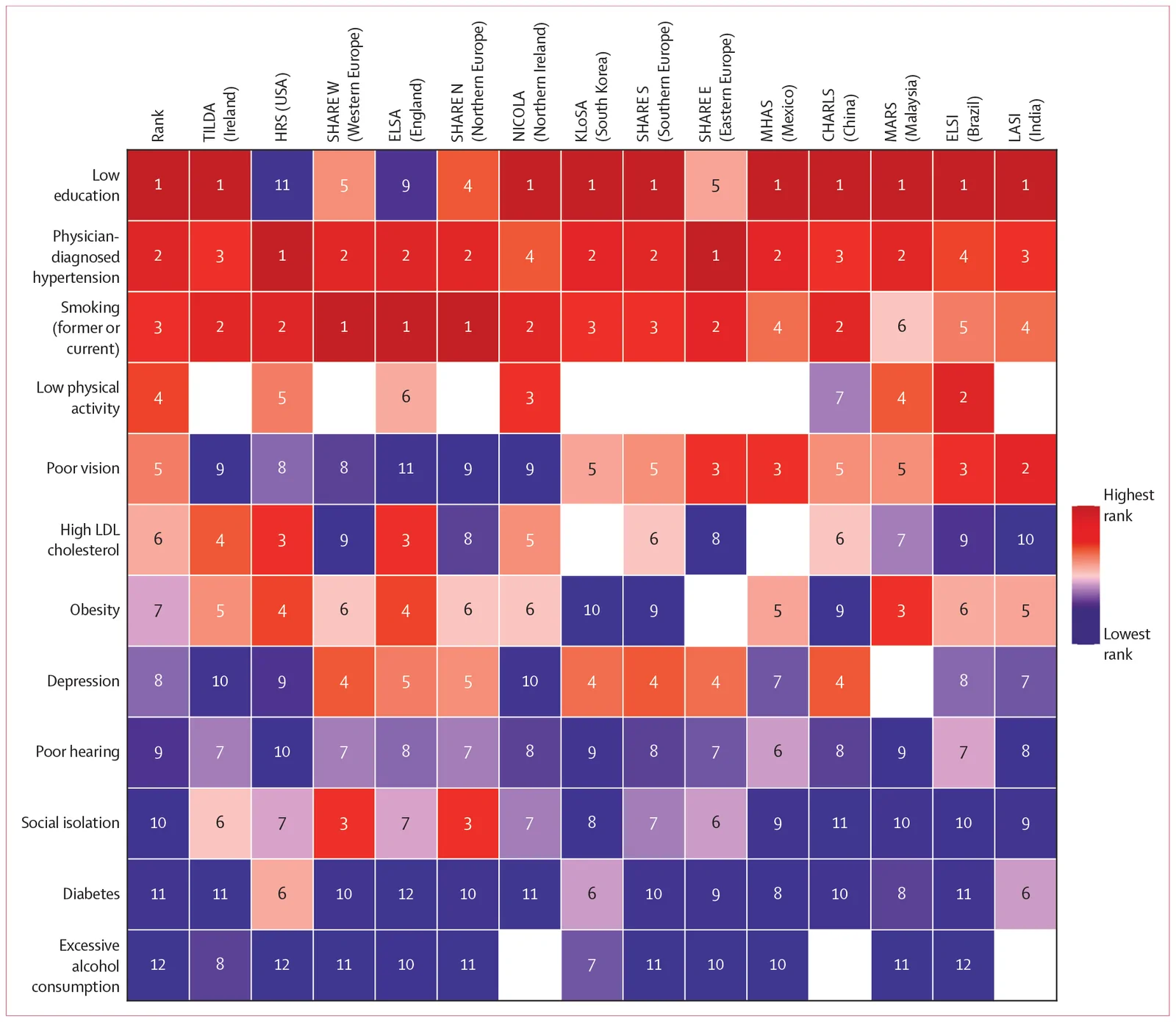
Heatmap of risk factor prevalence across countries and regions The overall ordering of risk factors is based on their average prevalence across settings. Colours represent within-country ranking, scaled to the number of risk factors available in each country. For this study, we considered education below the level of secondary education as low education. Physical activity was quantified using the Leisure Score Index. CHARLS=China Health and Retirement Longitudinal Study. E=East. ELSA=English Longitudinal Study of Ageing. ELSI=Brazilian Longitudinal Study of Ageing. HRS=Health and Retirement Study. KLoSA=Korean Longitudinal Study of Ageing. LASI=Longitudinal Ageing Study in India. MARS=Malaysia Ageing and Retirement Survey. MHAS=Mexican Health and Aging Study. N=North. NICOLA=Northern Ireland Cohort for the Longitudinal Study of Ageing. S=South. SHARE=Survey of Health, Ageing and Retirement in Europe. TILDA=The Irish Longitudinal Study on Ageing. W=West.

Examining the rank order of risk factor prevalence across countries further emphasised cross-national patterns (figure 1; appendix pp 30-31). Low education (first rank in nine of 14 countries or regions), hypertension (rank ≤4 in all countries or regions), and smoking (rank ≤4 in 12 of 14 countries or regions) were among the most prevalent risk factors in most settings. Nevertheless, low education was less prevalent in HICs, whereas smoking showed the opposite pattern. Social isolation, high cholesterol, and obesity had higher ranks in HICs, whereas poor vision had a higher rank in many LMICs. There were a few notable exceptions to the general pattern of contrasting risk factor prevalence in HICs and LMICs. For example, excessive alcohol consumption was the seventh-ranked risk in KLoSA (South Korea) and the eighth-ranked risk in TILDA (Ireland), whereas it ranked lower (ranging from 10^th^ to 12^th^ ranking) in all other contexts. In addition to excessive alcohol consumption, social isolation (in LMICs) and diabetes were among the lowest ranked factors in many countries.

**Figure 2:**
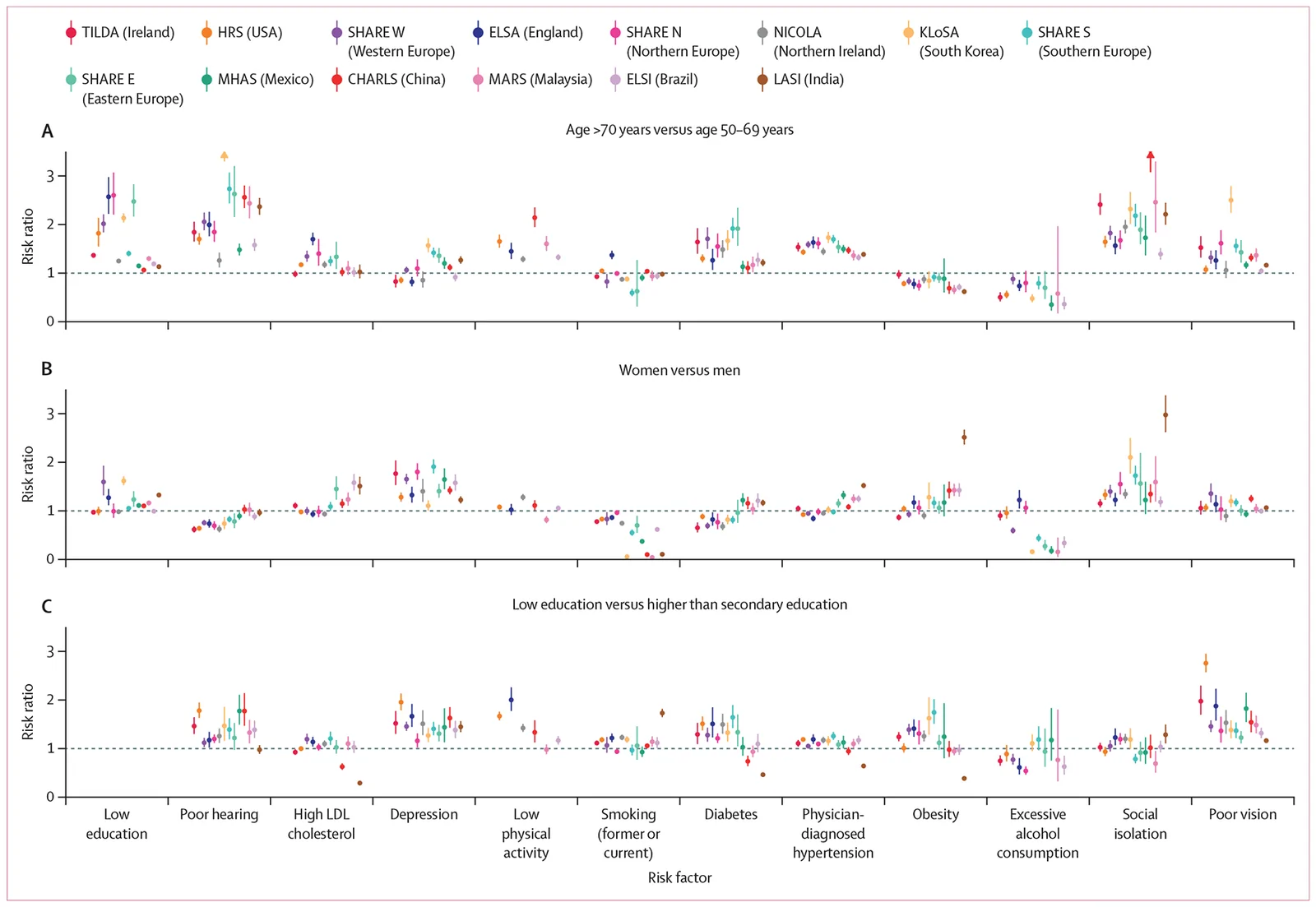
Comparative patterns of risk factors by (A) age, (B) gender, and (C) education across countries and regions Risk ratios were estimated using country-specific and risk factor-specific Poisson models with robust standard errors. Models were mutually adjusted for all three factors. For this study, we considered education below the level of secondary education as low education. Physical activity was quantified using the Leisure Score Index. CHARLS=China Health and Retirement Longitudinal Study. E=East. ELSA=English Longitudinal Study of Ageing. ELSI=Brazilian Longitudinal Study of Ageing. HRS=Health and Retirement Study. KLoSA=Korean Longitudinal Study of Ageing. LASI=Longitudinal Ageing Study in India. MARS=Malaysia Ageing and Retirement Survey. MHAS=Mexican Health and Aging Study. N=North. NICOLA=Northern Ireland Cohort for the Longitudinal Study of Ageing. S=South. SHARE=Survey of Health, Ageing and Retirement in Europe. TILDA=The Irish Longitudinal Study on Ageing. W=West.

Patterns by demographic variables showed that adults aged 70 years and older were more likely to have most risk factors across settings, except for smoking, obesity, and excessive alcohol consumption (figure 2; appendix pp 32-34). Additionally, age differences in low education were greater in HICs than in LMICs. For example, older adults in England were 157% (95% CI 122–198) more likely to have low education, whereas older adults in Malaysia were 30% (95% CI 25–35) more likely to have low education. Cardiovascular disease risk factors were more common in men than in women in HICs but more common in women than men in LMICs. For example, women were 35% (95% CI 24–44) less likely to have diabetes than men in TILDA (Ireland) but 21% (95% CI 7–36) more likely to have diabetes than men in ELSI (Brazil). The gender difference in poor hearing, with higher prevalence in men, was also greater in HICs. In contrast, behavioural risk factors (smoking and alcohol consumption) were more common in men only in LMICs. Across almost all (13 of 14) countries and regions, symptoms of depression and social isolation were more common in women. Many risk factors were more common in those with low education, with significantly higher prevalence among those with low education for at least half of the countries or regions with data for eight of 11 of included risk factors. Exceptions in some countries included cardiovascular risk factors, namely high cholesterol, diabetes, hypertension, and obesity, which were more common in those with high education in LASI (India) and CHARLS (China; only for cholesterol and diabetes). Physical inactivity co-occurred with low education in HICs, but this pattern was less apparent in LMICs.

Education gradients differed by gender for many risk factors (appendix p 35). For example, education gradients were larger in women for several cardiovascular disease risk factors, including diabetes, hypertension, and obesity, across most countries and regions.

**Figure 3:**
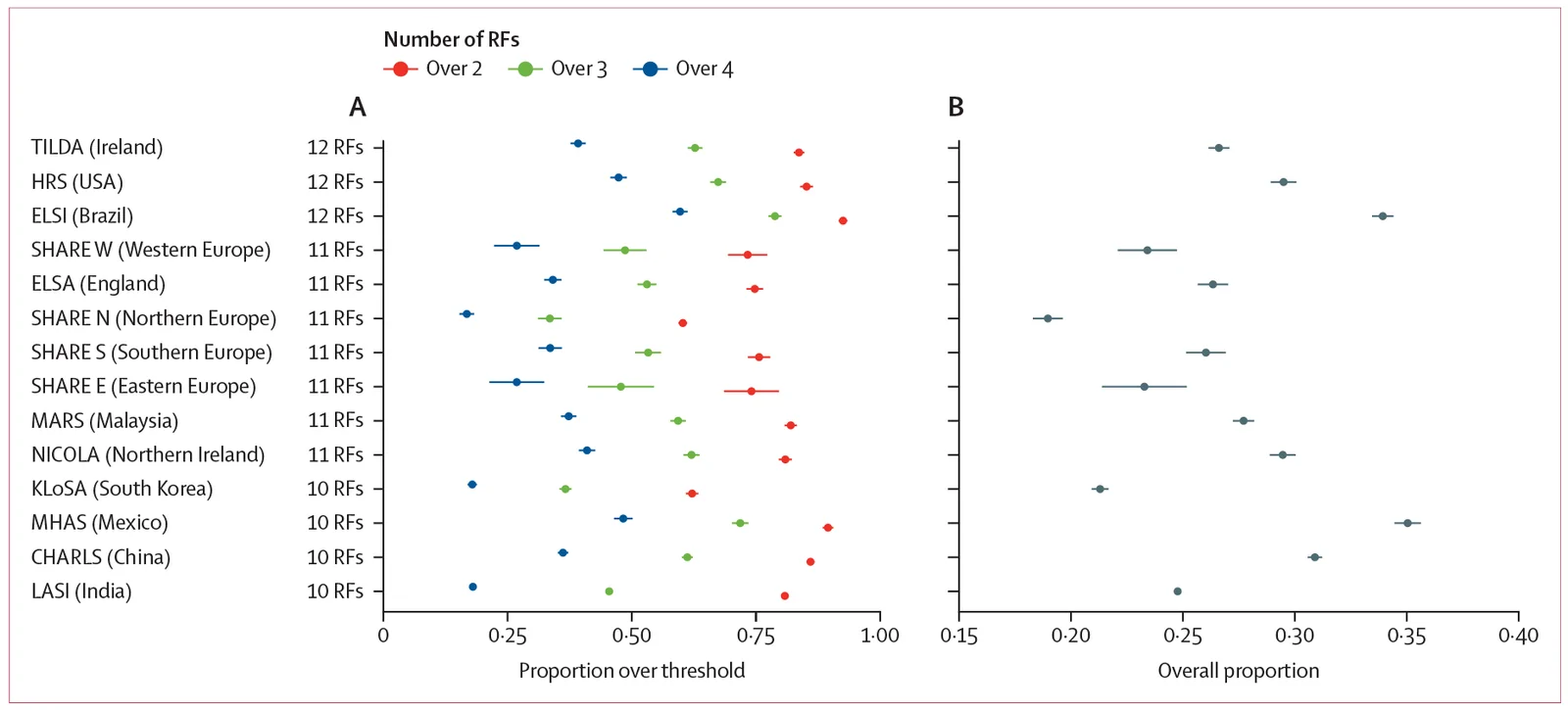
Patterns of co-occurring risk factors across countries and regions (A) The proportion of individuals with two or more risk factors. (B) The average proportion of risk factors present across respondents in each country or region after accounting for differences in the number of available risk factors across studies. CHARLS=China Health and Retirement Longitudinal Study. E=East. ELSA=English Longitudinal Study of Ageing. ELSI=Brazilian Longitudinal Study of Ageing. HRS=Health and Retirement Study. KLoSA=Korean Longitudinal Study of Ageing. LASI=Longitudinal Ageing Study in India. MARS=Malaysia Ageing and Retirement Survey. MHAS=Mexican Health and Aging Study. N=North. NICOLA=Northern Ireland Cohort for the Longitudinal Study of Ageing. RF=risk factor. S=South. SHARE=Survey of Health, Ageing and Retirement in Europe. TILDA=The Irish Longitudinal Study on Ageing. W=West.

**Figure 4:**
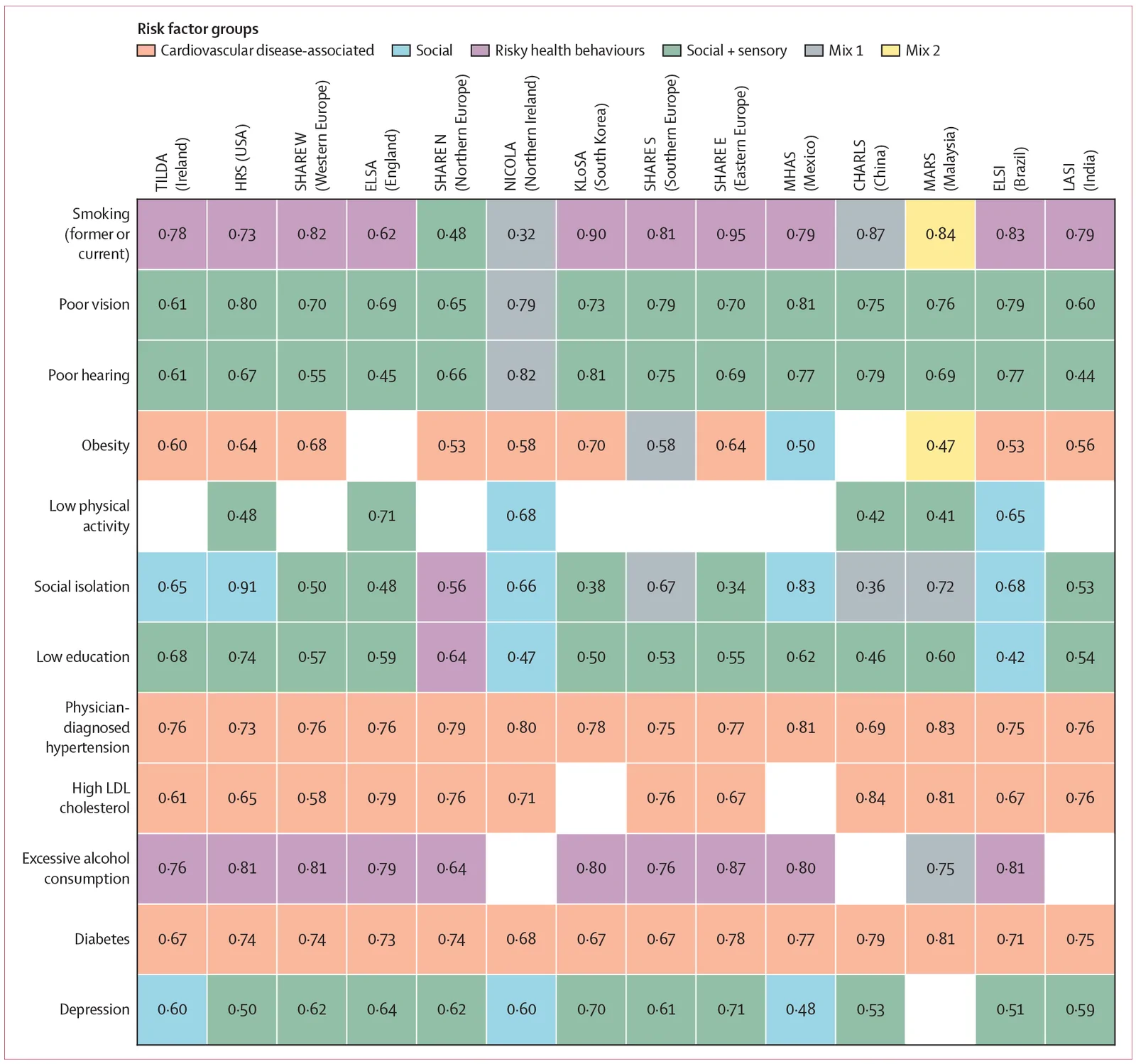
Clusters of risk factors across countries and regions Parallel analysis was used to identify the number of clusters, and principal component analysis was used with modal assignment to assign risk factors to clusters based on the highest factor loadings. Values represent standardised loadings of risk factors (scale range 0–1). Higher values indicate stronger associations of the risk factors with the cluster. Mix 1 and Mix 2 denote heterogeneous clusters that varied across countries and represent less common, inconsistent combinations of risk factors. For this study, we considered education below the level of secondary education as low education. Physical activity was quantified using the Leisure Score Index. CHARLS=China Health and Retirement Longitudinal Study. E=East. ELSA=English Longitudinal Study of Ageing. ELSI=Brazilian Longitudinal Study of Ageing. HRS=Health and Retirement Study. KLoSA=Korean Longitudinal Study of Ageing. LASI=Longitudinal Ageing Study in India. MARS=Malaysia Ageing and Retirement Survey. MHAS=Mexican Health and Aging Study. N=North. NICOLA=Northern Ireland Cohort for the Longitudinal Study of Ageing. S=South. SHARE=Survey of Health, Ageing and Retirement in Europe. TILDA=The Irish Longitudinal Study on Ageing. W=West.

Risk factors frequently co-occurred across all settings, with the prevalence of at least two risk factors exceeding 50% in all countries and regions (figure 3). In most (11 of 14) countries and regions, the prevalence of at least four risk factors also exceeded 20%. Estimates of the average number of co-occurring risk factors of the total number of risk factors measured (ie, a proportion, scaled from 0 to 1, representing the burden of co-occurring risk factors) for each country or region showed the highest levels of co-occurring risk factors in the two Latin American studies (MHAS [35%, 95% CI 34–36; Mexico] and ELSI [34%, 95% CI 33–34; Brazil]), followed by CHARLS (31%, 95% CI 31–31; China), HRS (29%, 95% CI 29–30; the USA), and NICOLA (29%; 95% CI 29–30; Northern Ireland). Risk factor clusters showed similarities across contexts (figure 4). A cardiovascular disease cluster, including diabetes, high cholesterol, hypertension, and in some cases obesity, was present across all contexts. Smoking and alcohol use also frequently co-occurred in the same cluster; poor hearing, poor vision, and low education co-occurred frequently as well.

Sensitivity analyses showed that the observed relative cross-country comparisons in primary analyses were largely similar across alternative specifications of binary or continuous measures of risk factors (appendix pp 18-29). Hypertension prevalence based on measured blood pressure or self-reported medication use was consistently higher than prevalence based on self-reported physician diagnosis; the difference ranged from 19% higher in CHARLS (China) to 71% higher in NICOLA (Northern Ireland). However, the rank ordering of hypertension prevalence across countries remained more stable across different hypertension measurement methods, with an average absolute rank difference of 1·33 (appendix pp 18-29). No pattern was observed in the magnitude of self-report bias (the difference between self-reported and measured hypertension prevalence) by GDP; the Pearson correlation coefficient between log(GDP) and prevalence difference was 0·23 (p=0·55).

## Discussion

Prevalence of the 12 selected dementia risk factors varied across settings, although there were some consistent patterns across HICs and LMICs. Low education (particularly in LMICs), hypertension, and smoking were among the most prevalent risk factors in most settings. Although patterns stratified by age, gender, and education showed substantial heterogeneity, similarities were observed among countries at similar levels of development. Despite cross-country differences in risk factor prevalence, risk factors co-occurred and clustered together in a similar manner. These patterns can inform strategic design choices that leverage commonality across countries to design intervention components or features meant to be shared across settings, while also highlighting areas where the customisation of interventions or policies aimed at dementia prevention are needed.

Low education was a priority risk factor in this analysis and in previous analyses that focused on PAFs.^2,3,6^ However, for risk factors other than low education, the prevalence rankings observed in this study differed from those based on PAFs, as previous studies considered both risk factor prevalence (the only focus of our study) and each risk factor’s effect on dementia. For example, although many analyses reported a high PAF for hearing loss,^3,6^ a low rank was observed for hearing loss in our study when examining prevalence across countries; this low rank might be partly attributable to our focus on prevalence rather than PAFs. Although PAFs provide a more appropriate metric for ranking overall importance because they consider both prevalence and effect size, comparisons of risk factor prevalence and patterns provide important information for planning and designing interventions or policies as they shape potential population-level impact and intervention feasibility. Additionally, our focus on prevalence enabled the inclusion of a broader range of countries without requiring data on dementia or assumptions of effect sizes being consistent across settings.

Including more wide-ranging country contexts than in previous cross-country analyses of dementia risk factors^6^ makes it easier to identify differences and similarities across countries and patterns by development levels. Similarities within HICs and LMICs in the prevalence and ranking of different risk factors suggest that insights from countries with similar development levels might be informative when designing interventions for dementia risk factors in settings with no empirical data. For example, low education is often the most prevalent risk factor in LMICs and might also influence other downstream risk factors,^23,24^ emphasising the importance of addressing low education in LMIC settings and other similar contexts. Estimates can also be compared with previous studies collating risk factor data across countries (eg, for the Western Pacific region),^25^ although other studies might provide poor-quality estimates based on models with sparse input data. New efforts to expand the availability of harmonised data sources are needed to improve data quality.

Methodological reasons for patterns should also be considered. Although cardiovascular disease risk factors were more prevalent in HICs, observed gradients were likely influenced by low or poor awareness of risk factors in LMICs or poor health-care access given surveys collected data on physician diagnoses. The low rank of diabetes prevalence compared to other risk factor prevalence across most settings might also be partly attributed to poor awareness of diabetes status. In addition to identifying patterns across countries, exceptions and potential outliers should be considered. For example, although obesity ranked high (between fourth and sixth) in most HICs, its prevalence was only 6·2% in South Korea. An understanding of country-specific contexts, including the social, political, and community structures in place, can help to identify outliers of broader patterns by development level and support tailoring of policies and interventions using frameworks for intervention adaptation.^26^

We also observed differences between HICs and LMICs in the patterns of risk factors by age, gender, and education. For example, women were less likely than men to engage in risky health behaviours such as smoking or alcohol use in LMICs, whereas this gender difference was smaller in HICs. Where evidence indicated substantial gender differences, findings can inform gender-specific intervention strategies and maximise the effects of available resources. Additionally, individuals with low education were more likely to have low physical activity in HICs, whereas this association was weaker in LMICs.

Patterns by gender and education highlight the ways in which exposure to dementia risk factors is shaped by broader social and societal contexts rather than solely by individual-level decision-making. Facets of identity and social or economic status (eg, age, gender, and education) influence an individual’s interactions with employers, community and government services, and health-care systems, which in turn affect the social, economic, and health factors associated with dementia risk. These patterns also emphasise how interventions that target poverty or broader sociodemographic inequalities might have sizeable downstream effects on late-life health outcomes such as dementia.^27^

Findings on the co-occurrence and clustering of dementia risk factors align with those from a previous analysis done in Denmark, which reported that 82% of respondents had more than one of 16 risk factors (in our study, this value is 62–93% across 10–12 risk factors).^10^ Although our data suggest that the average number of co-occurring risk factors varied across countries and regions (from 21% in Korea to 35% in Mexico), findings generally emphasised the importance of a holistic approach to dementia prevention across the range of established risk factors. The low effectiveness of many single-factor interventions might partly reflect failure to address multiple co-occurring risk factors.^28^ Risk factors might co-occur for many reasons, including biological links via mechanistic pathways (eg, cardiovascular disease risk factors), shared upstream determinants (eg, risky behaviours or socioeconomic factors), or mediating mechanisms (eg, education influencing access to correction for sensory impairment). In all cases, the co-occurrence and clustering of risk factors could guide efforts to target multiple risk factors that commonly cluster together; however, the reason behind clustering could influence design choices. For example, if multiple risk factors lie on the same biological pathway, targeting the most upstream factor might be most effective. Although multidomain interventions that include various components, such as personalised guidance on diet and physical activity, cognitive training, and increased social engagement, are highly effective,^13,14,29^ the effects of such interventions might not persist over the long term.^30^ Cluster-tailored multidomain trials would help to evaluate whether cluster-informed intervention designs can improve intervention response.

Strengths of this study include the large number of countries and regions, the inclusion of population-based samples to ensure generalisability of findings, the use of harmonised data to maximise comparability between contexts, and the in-depth analysis of risk factor prevalence and patterns. Limitations of the study include the analysis being restricted to the dementia risk factors reported in the *Lancet* Commission report; although these risk factors were selected based on evidence-based approaches with sufficient epidemiological support,^3^ future updates should consider additional risk factors as evidence emerges. We also used binary versions of all risk factors to simplify the interpretation of findings and comparisons, although, continuous measures (eg, pack-years of smoking) can provide more detailed information if available. Furthermore, despite efforts to maximise harmonisation during study design, data preparation, and data analysis, inconsistency remained. For example, survey questions on alcohol use relied on different response options across settings, and available data from the LASI survey in India did not include comparable alcohol consumption-related questions. In some cases, inconsistency reflects necessary cultural adaptations to ensure that questions are interpreted correctly and remain relevant to populations of interest. In other cases, despite the use of identical survey items, factors related to study context might influence comparisons. For example, although all surveys included questions on physician diagnosis of diabetes or hypertension, differences in health-care access and disease awareness across countries complicate comparisons. We compared self-reported and objective measures of hypertension to assess this bias, given the availability of objective measures across many surveys. Although we did not observe a strong association of self-report bias and GDP for hypertension, previous single-setting studies have reported higher self-report bias in groups with lower socioeconomic status, raising concerns of differential bias.^31-33^ Similar bias patterns might occur for diabetes as well.^32^ More standardised implementation of objective biomarker measurement protocols across additional surveys and conditions would help to improve such measurements. Self-report bias in other risk factors and survival bias should also be considered, especially given that self-reports of life course risk factors are based on cross-sectional data of respondents in mid to late life. Moreover, efforts to harmonise data across studies also led to the oversimplification of some risk factor definitions in our study. For example, we used living alone as an indicator of social isolation, simplifying a complex construct to enable harmonised measurement, and we acknowledge that cultural differences in multi-generational living could alter the interpretation of this indicator despite consistent measurement. Although rates of missing data were generally low, with instances of higher missing data explained by study design features, potential bias can arise from excluding respondents with missing data. Additionally, despite our efforts to balance the competing priorities of using the most up-to-date information with representativeness by selecting the most recent wave with a refresher sample, some data were outdated (eg TILDA in Ireland; 2009–10). Regular refresher sampling would ensure the availability of timely, population-representative data and enhance the utility of comparative analyses such as those in our study; however, such efforts require substantial investment. Future iterations of our study should include emerging data on additional risk factors (eg, sleep^34^) and new country contexts and should consider examining temporal changes in risk factors.

Our findings provide important insights into differences in the prevalence and patterns of dementia risk factors across contexts and can inform the optimal design of interventions and policies to address modifiable dementia risk factors. Although the development of national dementia plans and individual-level recommendations and guidelines is a useful initial step, such approaches often do not result in behavioural changes in individuals.^35,36^ Population-level approaches focused on implementing policy changes might be more effective and produce substantial cost savings for health systems.^37,38^ Although differences across contexts likely preclude a one-size-fits-all approach, similarities by development level suggest that policy makers can draw on evidence from countries with comparable contexts to inform adaptation. Moreover, areas of commonality, including the high levels of co-occurring risk factors and similar risk factor clusters, can be leveraged as components of a universal design strategy that can be adapted to specific contexts.^26^ The availability of harmonised data, such as those analysed here, is essential for generating cross-national insights and supporting the development of context-specific solutions in participating countries. Thus, our findings highlight opportunities to ensure that efforts to prevent or delay dementia are based on high-quality quantitative evidence, leading to effective interventions with maximum benefits.

## Supplementary Material

1

## Figures and Tables

**Table:** Weighted prevalence of 12 risk factors for dementia across 14 geographical countries and regions

	TILDA	HRS	SHARE W	ELSA	SHARE N	NICOLA	KLoSA	SHARE S	SHARE E	MHAS	CHARLS	MARS	ELSI	LASI	Cross-national challenges*
															
	Ireland	USA	Western Europe	England	Northern Europe	Northern Ireland	South Korea	Southern Europe	Eastern Europe	Mexico	China	Malaysia	Brazil	India	
Low education	63·4%	12·0%	24·6%	20·4%	26·4%	62·5%	47·7%	69·5%	34·0%	84·0%	85·6%	70·0%	76·4%	76·7%	None identified
Hearing loss	15·2%	19·4%	20·4%	21·0%	17·7%	24·0%	7·1%	20·8%	23·0%	33·3%	14·9%	17·0%	26·4%	7·4%	Low awareness of risk factor
High LDL cholesterol	37·6%	53·1	19·9%	41·1%	17·6%	36·4%	··	27·1%	21·6%	··	23·0%	25·2%	23·0%	2·9%	Low awareness of risk factor
Depression	9·8%	19·8%	28·1%	25·1%	19·6%	14·1%	23·5%	32·2%	37·6%	30·7%	37·2%	··	26·0%	12·9%	Interpretation
Physical inactivity	··	27·8%	··	24·5%	··	43·0%	··	··	··	··	15·4%	31·9%	52·3%	··	Interpretation
Smoking	57·1%	53·8%	58·9%	53·0%	54·3%	51·6%	33·4%	40·5%	53·2%	37·3%	44·0%	25·8%	None identified	19·8%	None identified
Diabetes	8·0%	22·6%	14·5%	12·9%	11·5%	11·3%	15·9%	17·6%	16·8%	26·1%	13·4%	23·0%	17·7%	13·1%	Low awareness of risk factor
Hypertension	37·8%	55·0%	46·7%	41·8%	44·1%	40·6%	36·6%	50·1%	54·3%	53·1%	40·2%	43·4%	49·0%	28·5%	Low awareness of risk factor
Obesity	35·3%	44·9%	21·3%	32·2%	18·2%	35·5%	6·2%	18·2%	29·4%	35·7%	14·5%	36·1%	30·7%	13·3%	None identified
Alcohol consumption	13·6%	8·0%	12·4%	16·8%	10·5%	··	13·8%	7·9%	5·4%	3·7%	··	0·6%	4·1%	··	None identified
Social isolation	22·9%	22·3%	29·7%	22·6%	33·4%	26·1%	13·1%	23·2%	23·2%	10·8%	10·0%	4·9%	21·2%	3·9%	None identified
Vision loss	11·0%	21·0%	20·2%	15·7%	16·4%	15·4%	17·8%	31·4%	38·8%	47·2%	30·4%	30·8%	49·6%	69·7%	Low awareness of risk factor

All data are in percentages. The regions are ordered by per capita gross domestic product (high->low) from left to right. CHARLS=China Health and Retirement Longitudinal Study. E=East. ELSA=English Longitudinal Study of Ageing. ELSI=Brazilian Longitudinal Study of Ageing. HRS=Health and Retirement Study. KLoSA=Korean Longitudinal Study of Ageing. LASI=Longitudinal Ageing Study in India. MARS=Malaysia Ageing and Retirement Survey. MHAS=Mexican Health and Aging Study. N=North. NICOLA=Northern Ireland Cohort for the Longitudinal Study of Ageing. S=South. SHARE=Survey of Health, Ageing and Retirement in Europe. TILDA=The Irish Longitudinal Study on Ageing. W=West.

* Cross-national challenges identify potential methodological or measurement reasons that could explain observed differences across countries or regions.

## Data Availability

Harmonised data from the Gateway to Global Ageing Data platform are available on study-specific websites. Data access across surveys typically requires users to register, provide an email address, and sign a data-use agreement. Additional details and instructions on accessing data from each survey are available at Gateway to Global Ageing Data platform.

## Research in context

### Evidence before this study

We searched PubMed on Dec 15, 2025, for studies on dementia risk factors using the terms “dementia”, “risk factor”, and (“global” or “international” or “cross-country”), without language restrictions, and also screened the reference lists of relevant articles to identify additional eligible studies. Few studies presented primary data on multiple risk factors across diverse contexts. A previous study reported population attributable fractions (PAFs) for dementia in Latin America, India, and China, but data were restricted to small geographical areas within these regions, and analyses focused on the overall PAF measure rather than examining patterns of risk factor prevalence in detail.

### Added value of this study

Our study extends previous small-scale efforts by analysing harmonised data (N=214 251) from population-based studies across 14 countries and regions to explore patterns of risk factor prevalence in greater detail. Using these unique data, we assessed the prevalence and rank order of risk factors, demographic patterns, and risk factor co-occurrence and clustering in diverse settings. Owing to the large number of countries and regions included, we improved on previous efforts by performing cross-country comparisons and identifying both common patterns and context-specific differences in dementia risk factors across development levels.

### Implications of all the available evidence

The findings highlight the value of generating cross-country comparative evidence, while also presenting the associated challenges. Similarities across settings with similar development levels can be leveraged to guide the adaptation of existing interventions when country-specific data do not exist. Moreover, consistency in patterns of risk factor co-occurrence can inform universal design frameworks that leverage elements of a common structure while ensuring flexibility and adaptability across diverse settings. Although a one-size-fits-all approach would not succeed, general patterns, combined with country-specific data (when available), can be used to ensure that the design of dementia policies and interventions is based on high-quality quantitative evidence. Future research should prioritise improving the availability of harmonised, high-quality data across diverse populations and incorporate standardised measurement approaches to strengthen cross-country comparisons.
