# Supplementary material for: Differences in the prevalence and patterns of dementia risk factors across 14 countries and regions: a harmonised cross-national analysis
Source: Lancet Healthy Longev. Author manuscript; Available in PMC 2026 Aug 24. (PMC13502513; doi:10.1016/j.lanhl.2026.100867)
Supplement: Supplementary file 1 [file NIHMS2198887-supplement-1.pdf]

THE LANCET
Healthy Longevity

Supplementary appendix

This appendix formed part of the original submission and has been peer reviewed. We post it as supplied by the authors.

Supplement to: Nichols E, Kunicki ZJ, Markot M, et al. Differences in the prevalence and patterns of dementia risk factors across 14 countries and regions: a harmonised cross-national analysis. *Lancet Healthy Longev* 2026. https://doi.org/10.1016/j.lanhl.2026.100867

Appendix

Table of Contents

Appendix 1: Map of included studies 3

Appendix 2: Study specific details 4

Appendix 3: Risk factor definitions 6

Education: 6

Poor Hearing: 6

High Cholesterol: 7

Depression using CESD or EURO-D: 7

Physical Inactivity: 9

Smoking: 10

Diabetes: 11

Hypertension: 11

Obesity: 12

Alcohol: 13

Social Isolation: 15

Poor Vision: 16

Appendix 4: Weight and survey design specifications 17

Appendix 5: Alternative risk factor specifications 18

Education 18

Hearing loss 19

High Cholesterol 20

Depressive symptoms 21

Physical Inactivity 22

Smoking 23

Diabetes 24

Hypertension 25

High BMI 26

Alcohol Consumption 27
Social Isolation 28
Poor Vision 29
Appendix 6: Confidence intervals for prevalence estimates 30
Appendix 7: Patterns in risk factor prevalence by demographic variables 32
Age 32
Gender 33
Education 34
Appendix 8: Gender-Education Interactions 35

Appendix 1: Map of included studies

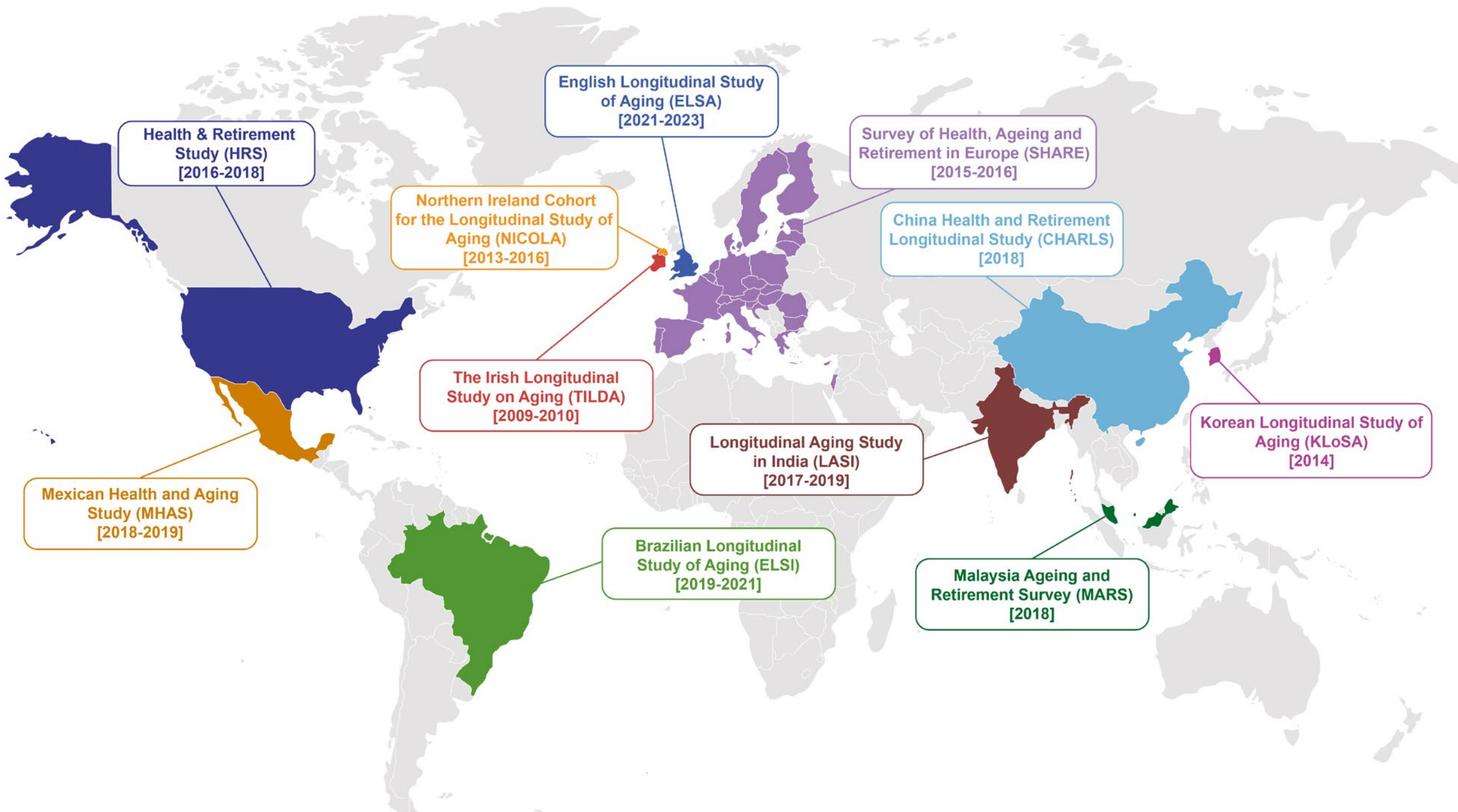

Figure S1.1. Map of included studies. Years indicate the dates of the most recent available survey round with a refresher sample in the Gateway to Global Aging harmonized datasets used throughout included analyses.

Appendix 2: Study specific details

Table S2.1. Names, locations, and design features of 11 included studies.

Variable	TILDA	HRS	SHARE	ELSA	NICOLA	KLoSA	MHAS	CHARLS	MARS	ELSI	LASI
Location	Ireland	United States	28 European countries & Israel	England	Northern Ireland	South Korea	Mexico	China	Malaysia	Brazil	India
Year started	2009	1992	2004	2002	2013	2006	2001	2011	2018	2015	2017
Years of primary wave used in present analysis	2009-10	2016-18	2015-16	2021-23	2013-16	2014	2018-19	2018	2018	2019-21	2017-19
Sample size in primary wave	8,163	19,623	64,840	6,226	8,280	7,949	15,857	16,141	4,057	9,949	53,166
# Waves in harmonized data	3	15	8	9	2	8	5	4	1	2	1
Sample size (min, max across waves)	5,713, 8,504	12,652, 22,034	13,948, 69,447	8,445, 12,099	6,097, 8,478	6,488, 10,254	13,704, 17,114	17,708, 21,097	5,612	9,412, 9,949	73,408
Age eligibility**	50+	51+	50+	50+	50+	45+	50+	45+	40+	50+	45+
Age, M (SD)	65.2 (9.3)	67.0 (11.4)	67.1 (10.3)	66.2 (10.5)	66.0 (10.2)	66.2 (10.9)	63.5 (11.1)	59.6 (10.5)	57.2 (10.8)	65.0 (10.2)	57.9 (11.7)
Women (%)	55.6%	56.0%	55.5%	54.5%	55.2%	56.2%	56.1%	51.8%	55.8%	57.9%	57.6%

* Demographic summaries are unweighted and based on pooled data across all waves with harmonized data available. **Though age eligibility varies across studies, in the present analysis we restricted all studies to respondents 50 years of age and older.

Additional information can be found on the Gateway to Global Aging website (https://g2aging.org/), and in cohort profile papers:

- TILDA: Donoghue OA, McGarrigle CA, Foley M, Fagan A, Meaney J, Kenny RA. Cohort Profile Update: The Irish Longitudinal Study on Ageing (TILDA). Int J Epidemiol 2018; **47**: 1398–1398l.
- HRS: Sonnega A, Faul JD, Ofstedal MB, Langa KM, Phillips JW, Weir DR. Cohort Profile: the Health and Retirement Study (HRS). International Journal of Epidemiology 2014; **43**: 576–85.

- ELSA: Steptoe A, Breeze E, Banks J, Nazroo J. Cohort profile: the English longitudinal study of ageing. Int J Epidemiol 2013; 42: 1640–8.; Zaninotto P, Steptoe A. English Longitudinal Study of Ageing. In: Encyclopedia of Gerontology and Population Aging. Springer, Cham, 2021: 1671–8.
- NICOLA: Neville C, Burns F, Cruise S, et al. Cohort Profile: The Northern Ireland Cohort for the Longitudinal Study of Ageing (NICOLA). Int J Epidemiol 2023; **52**: e211–21.
- SHARE: Börsch-Supan A, Brandt M, Hunkler C, et al. Data Resource Profile: The Survey of Health, Ageing and Retirement in Europe (SHARE). Int J Epidemiol 2013; **42**: 992–1001.
- KLoSA: Shin C. Korean Longitudinal Study of Ageing. In: Encyclopedia of Gerontology and Population Aging. Springer, Cham, 2021: 2833–6.
- MHAS: Wong R, Michaels-Obregon A, Palloni A. Cohort Profile: The Mexican Health and Aging Study (MHAS). Int J Epidemiol 2017; **46**: e2.
- CHARLS: Zhao Y, Hu Y, Smith JP, Strauss J, Yang G. Cohort profile: the China Health and Retirement Longitudinal Study (CHARLS). Int J Epidemiol 2014; **43**: 61–8.
- MARS: Mansor N, Awang H, Rashid NFA. Malaysia Ageing and Retirement Survey. In: Encyclopedia of Gerontology and Population Aging. Springer, Cham, 2021: 3032–6.
- ELSI: Lima-Costa MF, de Melo Mambrini JV, Bof de Andrade F, et al. Cohort Profile: The Brazilian Longitudinal Study of Ageing (ELSI-Brazil). Int J Epidemiol 2022; **52**: e57–65.
- LASI: Perianayagam A, Bloom D, Lee J, et al. Cohort Profile: The Longitudinal Ageing Study in India (LASI). International Journal of Epidemiology 2022; **51**: e167–76.

Appendix 3: Risk factor definitions

Education:

Study	Wave	Years	Scale	Notes
TILDA	1	09-10	A	
HRS	13	2016	A	
SHARE	6	2015	A	
ELSA	10	21-23	A	
NICOLA	1	13-16	A	
KLoSA	5	2014	A	
MHAS	5	2018	A	
CHARLS	4	2018	A	
MARS	1	2018	A	
ELSI	2	19-21	A	
LASI	1	17-21	A	

Binary = 1.less than upper secondary, 0.upper secondary or greater than upper secondary

A = (0) High education: 2.upper secondary and vocational, 3.tertiary; (1) Low education: 1.less than upper secondary

Poor Hearing:

Study	Wave	Years	Scale	Notes
TILDA	1	09-10	A	
HRS	13	2016	A	
SHARE	6	2015	A	
ELSA	10	21-23	A	
NICOLA	1	13-16	A	
KLoSA	5	2014	B	Special case
MHAS	5	2018	C	
CHARLS	4	2018	A	Special case
MARS	1	2018	D	
ELSI	2	19-21	E	
LASI	1	17-21	F	

Binary = 1.fair or poor, 0.good, very good, or excellent

A = (0) Good hearing: 1.excellent, 2.very good, 3.good; (1) Poor hearing: 4.fair, 5.poor

- **CHARLS uses 1.poor, 0.excellent, very good, good, or fair**

B = (0) Good hearing: 1.very good, 2.good, 3.fair; (1) Poor hearing: 4.poor, 5.very poor

- **KLoSA uses 1.poor or very poor, 0.very good, good, or fair**

C = (0) Good hearing: 1.excellent, 2.very good, 3.good; (1) Poor hearing: 4.fair, 5.poor, 6.legally deaf

D = (0) Good hearing: 3.good; (1) Poor hearing: 4.fair, 5.poor

E = (0) Good hearing: 1.very good or excellent, 2.good; (1) Poor hearing: 3.fair, 4.bad, 5.very bad

F = Ever had hearing/ ear related problem? (0) Good hearing: 0.no, (1) Poor hearing: 1.yes

Alternative hearing measure: KLoSA and CHARLS use same scale as others with "fair" indicating poor hearing.

High Cholesterol:

Study	Wave	Years	Scale	Notes
TILDA	1	09-10	A	
HRS	13	2016	A	
SHARE	6	2015	A	
ELSA	10	21-23	A	
NICOLA	1	13-16	A	
KLoSA				
MHAS				
CHARLS	4	2018	A	Called dyslipidemia
MARS	1	2018	A	
ELSI	2	19-21	A	
LASI	1	17-21	A	

Binary = 1.yes, 0.no

A = Doctor ever diagnosed with high cholesterol? (0) No high cholesterol: 0.no; (1) High cholesterol: 1.yes

Depression using CESD or EURO-D:

Study	Wave	Years	Scale	Notes
TILDA	1	09-10	9/24 4-pt	1,2
HRS	13	2016	3/8 binary	3
SHARE	6	2015	4/12 binary	4, uses EURO-D
ELSA	10	21-23	3/8 binary	5,6
NICOLA	1	13-16	9/24 4-pt	same scale as TILDA

KLoSA	5	2014	10/30 4-pt	7,8
MHAS	5	2018	5/9 binary	9,10
CHARLS	4	2018	10/30 4-pt	11,12
MARS				
ELSI	2	19-21	3/8 binary	same scale as ELSA and HRS
LASI	1	17-21	15/30 4-pt	13

1. Mallett J, Redican E, Doherty AS, Shevlin M, Adamson G. Depression trajectories among older community dwelling adults: Results from the Irish Longitudinal Study on Ageing (TILDA). Journal of Affective Disorders 2022; **298**: 345–54.
2. Briggs R, Carey D, O'Halloran AM, Kenny RA, Kennelly SP. Validation of the 8-item Centre for Epidemiological Studies Depression Scale in a cohort of community-dwelling older people: data from The Irish Longitudinal Study on Ageing (TILDA). Eur Geriatr Med 2018; **9**: 121–6.
3. Soh Y, Tiemeier H, Kawachi I, Berkman LF, Kubzansky LD. Eight-Year Depressive Symptom Trajectories and Incident Stroke: A 10-Year Follow-Up of the HRS (Health and Retirement Study). Stroke 2022; **53**: 2569–76.
4. Courtin E, Knapp M, Grundy E, Avendano-Pabon M. Are different measures of depressive symptoms in old age comparable? An analysis of the CES-D and Euro-D scales in 13 countries. International Journal of Methods in Psychiatric Research 2015; **24**: 287–304.
5. Poole L, Steptoe A. Depressive symptoms predict incident chronic disease burden 10 years later: Findings from the English Longitudinal Study of Ageing (ELSA). Journal of Psychosomatic Research 2018; **113**: 30–6.
6. Garfield V, Llewellyn CH, Kumari M. The relationship between physical activity, sleep duration and depressive symptoms in older adults: The English Longitudinal Study of Ageing (ELSA). Preventive Medicine Reports 2016; **4**: 512–6.
7. Kyung LH, Hee LS. Depression, Diabetes, and Healthcare Utilization: Results from the Korean Longitudinal Study of Aging (KLoSA). Iran J Public Health 2014; **43**: 6–15.
8. Kim H, Kim SH, Cho YJ. Combined Effects of Depression and Chronic Disease on the Risk of Mortality: The Korean Longitudinal Study of Aging (2006-2016). Journal of Korean Medical Science 2021; **36**. DOI:10.3346/jkms.2021.36.e99.
9. Giraldo-Rodríguez L, Torres-Castro S, Roa-Rojas PA, Alvarez-Cisneros T. Demographic, socioeconomic and health determinants of depressive symptoms in adults 50 years and older from Mexico: a secondary data longitudinal analysis from the Mexican Health and Aging Study. BMJ Open 2024; **14**: e075035.
10. Gutierrez S, Wong R, Milani SA. The pain and depressive symptoms cascade: A bidirectional analysis of the Mexican Health and Aging Study 2012–2015. International Journal of Geriatric Psychiatry 2022; **37**. DOI:10.1002/gps.5812.
11. Luo H, Li J, Zhang Q, et al. Obesity and the onset of depressive symptoms among middle-aged and older adults in China: evidence from the CHARLS. BMC Public Health 2018; **18**: 909.
12. Zhou L, Ma X, Wang W. Relationship between Cognitive Performance and Depressive Symptoms in Chinese Older Adults: The China Health and Retirement Longitudinal Study (CHARLS). Journal of Affective Disorders 2021; **281**: 454–8.

13. Muhammad T, Lee S, Kumar M, Sekher TV, Varghese M. Agreement between CES-D and CIDI-SF scales of depression among older adults: a cross-sectional comparative study based on the longitudinal aging study in India, 2017-19. BMC Psychiatry 2025; **25**: 244.

CESD summary scores were created by taking the sum of depression questions. Some studies had binary yes/no questions and others had categorical 4-point scales (indicating frequency in the past week feeling depressed: 1.Rarely or none of the time (less than 1 day), 2.Some or a little of the time (1-2 days), 3.Occasionally or a moderate amount of the time (3-4 days), 4.All of the time (5-7 days). All questions are coded such a higher number represents greater depressive symptomology. If the individual has an equal depression summary score or higher as the scale then they are depressed (Ex. ELSA: 3 or more questions answered yes means they are assigned depression). SHARE uses the EURO-D depression scale with binary yes/no questions.

Cutoffs determined from literature. All 8 item binary scales used 3/8 as the cutoff.

Physical Inactivity:

The Leisure Score Index (LSI) was calculated using reported days of vigorous, moderate, and light physical activity per week. We used alternatives for studies that don't have all 3 levels of activity.

Study	Wave	Years	Scale	Notes
TILDA	1	09-10	A	
HRS	13	2016	B	
SHARE			C	(V,M) w6, 2015
ELSA	10	21-23	C	
NICOLA	1	13-16	A	
KLoSA			D	(V) w5, 2014
MHAS				
CHARLS	4	2018	A, D	
MARS	1	2018	B	
ELSI	2	19-21	A	
LASI			B	(V,M) w1 17-21

A = Vigorous, moderate, and light physical activity:

2. 2. >1 per week → 4.5 days per week – the average of potential options from 2-7 days = (2+7)/2
3. 1 per week → 1 day per week
4. 4.1-3 per month → 0.5 days per week
5. hardly ever or never → 0 days per week

B = Vigorous, moderate, and light physical activity:

1. every day → 7 days per week
2. >1 per week → 4 days per week – the average pf potential options from 2-6 days = (2+6)/2
3. 1 per week → 1 day per week
4. 1-3 per month → 0.5 days per week
5. never → 0 days per week

<u>Alternate way for turning categorical activity frequency of "more than once per week" into continuous.</u> Take the 4 studies (TILDA, CHARLS, NICOLA, ELSI) that have both continuous and categorical questions and calculate the average number of days per week respondents reported exercising, when the reported exercising "more than once per week". We pooled the data across these 4 studies and used this as the number of days per week instead of 4 as we used in the primary analysis.

C = continuous numbers of days exercised per week variable for each level of activity

Leisure Score Index (LSI) = (vigorous act * 9) + (moderate act * 5) + (light act * 3) (1)

Binary = 1.less than LSI of 14, 0.greater than or equal to LSI of 14. (1) (LSI less than 14 was defined in literature as insufficiently active; this is equivalent to 7 kcal/kg/week or 420 metabolic minutes/week).

1. Godin Leisure-Time Exercise Questionnaire. Medicine & Science in Sports & Exercise 1997; **29**: 36.
2. Godin G. The Godin-Shephard Leisure-Time Physical Activity Questionnaire. The Health & Fitness Journal of Canada 2011; **4**: 18–22.

<u>Alternatives for vigorous, moderate, and light activity alone:</u>

A = inactive for 4 and 5, active for 2 and 3

B = inactive for 4 and 5, active for 1, 2, and 3

C = inactive for 0 days, and active for 1 to 7

D = CHARLS uses question: Any vigorous physical activity or exercise for at least 10 minutes. Inactive for no, active for yes.

Smoking:

Study	Wave	Years	Scale	Notes
TILDA	1	09-10	A	
HRS	13	2016	A	
SHARE	6	2015	A	
ELSA	10	21-23	A	

NICOLA	1	13-16	A	
KLoSA	5	2014	A	
MHAS	5	2018	A	
CHARLS	4	2018	A	
MARS	1	2018	A	
ELSI	2	19-21	A	
LASI	1	17-21	A	

Binary = 1.has smoked ever or currently smokes, 0.does not currently smoke and has never smoked

A = Have you ever smoked? Do you currently smoke?

For SHARE, the current smoking status question was skipped for returning respondents; therefore, we brought forward Wave 5 responses for these respondents.

Diabetes:

Study	Wave	Years	Scale	Notes
TILDA	1	09-10	A	
HRS	13	2016	A	
SHARE	6	2015	A	
ELSA	10	21-23	A	
NICOLA	1	13-16	A	
KLoSA	5	2014	A	
MHAS	5	2018	A	
CHARLS	4	2018	A	
MARS	1	2018	A	
ELSI	2	19-21	A	
LASI	1	17-21	A	

Binary = 1.yes, 0.no

A = Has your doctor ever diagnosed you with diabetes? (0) No diabetes: 0.no; (1) Diabetes: 1.yes

Hypertension:

Study	Wave	Years	Scale	Notes
TILDA	1	09-10	A, B	w1, 09-10
HRS	13	2016	A, B	w13, 2016
SHARE	6	2015	A	
ELSA	10	21-23	A, B	W6, 12-13

NICOLA	1	13-16	A, B	w1, 13-16
KLoSA	5	2014	A	
MHAS	5	2018	A, B	w3, 2012
CHARLS	4	2018	A, B	w3, 2015
MARS	1	2018	A, B	w1, 2018
ELSI	2	19-21	A, B	w2, 19-21
LASI	1	17-21	A, B	W1, 17-21

Binary = 1.yes, 0.no

A = Has your doctor ever diagnosed you with hypertension? or Whether you currently take medication for high blood pressure?

<u>Secondary variable based on measured BP to determine hypertension:</u>

B = measured diastolic and systolic

Binary = 1.diastolic >= 90 OR systolic >=140 OR self-reported medication use, 0. diastolic < 90 AND systolic <140 (1)

Though the American Heart Association thresholds for hypertension were updated in 2017, we use the older thresholds in the current study given that these better align with the period of data collection across most studies and new guidelines can take time to be fully adapted. Further, international guidelines align better with the older AHA thresholds.

1. Evbayekha EO, Okobi OE, Okobi T, et al. The Evolution of Hypertension Guidelines Over the Last 20+ Years: A Comprehensive Review. Cureus; **14**: e31437.

Obesity:

Study	Wave	Years	Scale	Notes
TILDA	1	09-10	A	
HRS	13	2016	B	
SHARE	6	2015	C	
ELSA	6	12-13	C	
NICOLA	1	13-16	A	
KLoSA	5	2014	C	
MHAS	3	2012	B	Self Report: w5, 2018
CHARLS	3	2015	A	
MARS	1	2018	A	
ELSI	2	19-21	A	
LASI	1	17-21	A	

A = Measured height and weight.

B = Measured height and weight and also self report height and weight.

C = Self reported height and weight.

Calculate BMI using height and weight, prioritizing measured.

Binary = 1.BMI greater than or equal to 30, 0.bmi less than 30 for non-Asian countries (TILDA, HRS, SHARE, ELSA, NICOLA, MHAS, ELSI)

1. Komaroff M. For Researchers on Obesity: Historical Review of Extra Body Weight Definitions. J Obes 2016; **2016**: 2460285.

Binary = 1.BMI greater than or equal to 27.5, 0.bmi less than 27.5 for Asian countries (KLoSA, CHARLS, MARS, LASI)

1. WHO expert consultation. Appropriate body-mass index for Asian populations and its implications for policy and intervention strategies. The Lancet 2004; **363**: 157–63.

<u>For analyses requiring co-occurring risk factors we used self-report for MHAS to ensure all risk factors were measured in the same wave.</u>

Alcohol:

**Study**	**Wave**	**Years**	**Scale**	**Notes**
TILDA	1	09-10	A, F	
HRS	13	2016	B, F	
SHARE	6	2015	C, F	Last week not per week (binge = w5, 2013)
ELSA	10	21-23	C, F	Binge: w3, 06-07
NICOLA				
KLoSA	5	2014	D, F	
MHAS	5	2018	B, F	
CHARLS				
MARS	1	2018	D, F	
ELSI	2	19-21	B, F	
LASI			G	Only binge

A = Continuous drinks per week

B = Continuous drinks per day when drinks.

DAYSperWEEK

0. none or less than once a month → 0 days
1. one to several times a month → 0.577 days, calculated as 2.5 days per month multiplied by 12/52 (12 months over 52 weeks) to get days per week = (2.5*12)/52
2. one to several times a week → 2.5 days
3. most days of the week → 5.5 days
4. Every day of the week → 7 days

Multiply continuous drinks per day when drinks by DAYSperWEEK to get continuous drinks per week.

C = Continuous drinks per day when drinks and continuous days per week R drinks. Multiply together to get drinks per week continuous.

D = Categorical drinks per day when drinks and days drinking in past month.

DAYSperWEEK

0. does not drink → 0 days
1. once a month → 0.231 days, calculated as 1 day per month multiplied by 12/52 (12 months over 52 weeks) to get days per week = 1*(12/52)
2. 2 to 3 times a week → 2.5 days
3. 4 or more times a week → 5.5 days

Drinks per day when drinks categories:

0. 0 or does not drink → 0 drinks
1. 1 to 2 → 1.5 drinks
2. 3 to 4 → 3.5 drinks
3. 5 to 6 → 5.5 drinks
4. 7 to 9 → 8 drinks
5. >10 → 10 drinks

Multiply the calculated days per week and drinks per day together to get the continuous drinks per week.

E = Continuous drinks per day when drinks and categorical frequency of days drinking. Categorical frequency of days drinking per week (answer category → numeric translation):

0. None or doesn't drink → 0 days
1. 1 or a couple of days per year → 0.038 days, calculated as 2 days per year then divided by 52 weeks per year to get days per week = 2/52.
2. Once a month → 0.231 days, calculated as 1 day per month multiplied by 12/52 (12 months over 52 weeks) to get days per week = 1*(12/52).

3. 2 to 3 days a month → 0.577 days, calculated as 2.5 days per month and then multiplied by 12 months / 52 weeks to get days per week = 2.5*(12/52).
4. Once a week → 1 day
5. 2 to 3 days a week → 2.5 days
6. 4 to 6 days a week → 5.5 days
7. Daily → 7 days

Multiply the calculated days per week by the continuous drinks per day to get continuous drinks per week.

Binary = 1.15 or more drinks per week for men and 8 or more drinks per week for women, 1.14 or less drinks per week for men and 7 or less drinks per week for women (1)

1. Centers for Disease Control and Prevention (U.S.) "What is excessive alcohol use?" (2014)

<u>Binge drinking alternative</u>

F = drinks per day when drinks continuous (some calculated from categorical variables as described above)

G = Do you ever binge drink?

Binary = 1.5 or more drinks per day for men and 4 or more drinks per day for women, 1.4 or less drinks per day for men and 3 or less drinks per day for women (LASI uses a binary variable only, defined as having 5 or more drinks on at least one occasion over the last three months)

Social Isolation:

Study	Wave	Years	Scale	Notes
TILDA	1	09-10	A	
HRS	13	2016	A	
SHARE	6	2015	A	
ELSA	10	21-23	A	
NICOLA	1	13-16	A	
KLoSA	5	2014	A	
MHAS	5	2018	A	
CHARLS	4	2018	A	
MARS	1	2018	A	
ELSI	2	19-21	A	
LASI	1	17-21	A	

A = (0) Does not live alone: >1 person living in the household; (1) Lives alone: One person lives in the household

Binary = 1.Lives alone 0.Does not live alone

Poor Vision:

Study	Wave	Years	Scale	Notes
TILDA	1	09-10	A	
HRS	13	2016	B	
SHARE	6	2015	B	
ELSA	10	21-23	B	
NICOLA	1	13-16	B	
KLoSA	5	2014	B	Special case
MHAS	5	2018	B	
CHARLS	4	2018	A	Special case
MARS	1	2018	C	
ELSI	2	19-21	D	
LASI	1	17-21	E	

Binary = 1.fair or poor, or blind, 0.good, very good, or excellent

A = (0) Good vision: 1.excellent, 2.very good, 3.good, (1) Poor vision: 4.fair, 5.poor, 6.blind

- **CHARLS uses 1.poor or blind, 0.excellent, very good, good, or fair**

B = (0) Good vision: 1.very good, 2.good, 3.fair, (1) Poor vision: 4.poor, 5.very poor, 6.blind

- **KLoSA uses 1.poor or very poor, or blind, 0.very good, good, or fair**

C = (0) Good vision: 3.good, (1) Poor vision: 4.fair, 5.poor

D = (0) Good vision: 1.very good, 2.good, (1) Poor vision: 3.fair, 4.poor, 5.very poor

E = (0) Good vision: 1.excellent, 2.very good, 3.good, (1) Poor vision: 4.fair, 5.poor

F = (0) Good vision: 1.very good or excellent, 2.good, (1) Poor vision: 3.fair, 4.bad, 5.very bad

Some studies ask about the respondent’s self-rated eyesight in general and some ask separate questions about self-rated near eyesight and far eyesight. Respondents who were categorized as having poor vision for either near or far eyesight were classified as having poor vision.

<u>Alternative vision:</u> KLoSA and CHARLS use the same scale as others.

Appendix 4: Weight and survey design specifications

- TILDA: survey weights + strata + cluster ids
- HRS: survey weights + strata + cluster ids
- SHARE: survey weights + strata + cluster
- ELSA: survey weighs + strata + cluster
- NICOLA: no survey weights or design features available
- KLoSA: survey weights + strata
- MHAS: survey weights with no other design features
- CHARLS: survey weights with no other design features
- MARS: no survey weights or design features available
- ELSI: survey weights + strata
- LASI: survey weights with no other design features

Appendix 5: Alternative risk factor specifications

All summary tables account for available survey weights and survey design information. Tables also include information on sample size and the amount of missing data.

Education

**Education (%, (N), [miss, %miss])**	**Low Education**	**Less than Secondary Education**	**Secondary Education**	**Tertiary Education**
**TILDA**	63.4%, (8171), [4, 0.0%]	63.4%, (8171), [4, 0.0%]	18.1%, (8171), [4, 0.0%]	18.5%, (8171), [4, 0.0%]
**HRS**	12.0%, (20901), [10, 0.0%]	12.0%, (20901), [10, 0.0%]	50.4%, (20901), [10, 0.0%]	37.6%, (20901), [10, 0.0%]
**SHARE W**	24.6%, (21515), [0, 0.0%]	24.6%, (21515), [0, 0.0%]	48.4%, (21515), [0, 0.0%]	27.0%, (21515), [0, 0.0%]
**ELSA**	20.4%, (6864), [618, 8.3%]	20.4%, (6864), [618, 8.3%]	56.2%, (6864), [618, 8.3%]	23.5%, (6864), [618, 8.3%]
**SHARE N**	26.4%, (13092), [0, 0.0%]	26.4%, (13092), [0, 0.0%]	37.5%, (13092), [0, 0.0%]	36.0%, (13092), [0, 0.0%]
**NICOLA**	62.5%, (8214), [66, 0.8%]	62.5%, (8214), [66, 0.8%]	21.5%, (8214), [66, 0.8%]	15.9%, (8214), [66, 0.8%]
**KLoSA**	47.7%, (7949), [0, 0.0%]	47.7%, (7949), [0, 0.0%]	35.7%, (7949), [0, 0.0%]	16.7%, (7949), [0, 0.0%]
**SHARE S**	69.5%, (23850), [0, 0.0%]	69.5%, (23850), [0, 0.0%]	20.2%, (23850), [0, 0.0%]	10.3%, (23850), [0, 0.0%]
**SHARE E**	34.0%, (6581), [0, 0.0%]	34.0%, (6581), [0, 0.0%]	55.4%, (6581), [0, 0.0%]	10.6%, (6581), [0, 0.0%]
**MHAS**	84.0%, (15651), [232, 1.5%]	84.0%, (15651), [232, 1.5%]	3.7%, (15651), [232, 1.5%]	12.4%, (15651), [232, 1.5%]
**CHARLS**	85.6%, (17284), [0, 0.0%]	85.6%, (17284), [0, 0.0%]	11.6%, (17284), [0, 0.0%]	2.8%, (17284), [0, 0.0%]
**MARS**	70.0%, (4057), [0, 0.0%]	70.0%, (4057), [0, 0.0%]	22.4%, (4057), [0, 0.0%]	7.7%, (4057), [0, 0.0%]
**ELSI**	76.4%, (9781), [168, 1.7%]	76.4%, (9781), [168, 1.7%]	17.5%, (9781), [168, 1.7%]	6.1%, (9781), [168, 1.7%]
**LASI**	76.7%, (53166), [0, 0.0%]	76.7%, (53166), [0, 0.0%]	18.6%, (53166), [0, 0.0%]	4.6%, (53166), [0, 0.0%]

Hearing loss

Hearing (%, (N), [miss, %miss])	Hearing Base Scale	Hearing Alt Scale
**TILDA**	15.2%, (8175), [0, 0.0%]	15.2%, (8175), [0, 0.0%]
**HRS**	19.4%, (20885), [26, 0.1%]	19.4%, (20885), [26, 0.1%]
**SHARE W**	20.4%, (21469), [46, 0.2%]	20.4%, (21469), [46, 0.2%]
**ELSA**	21.0%, (7480), [2, 0.0%]	21.0%, (7480), [2, 0.0%]
**SHARE N**	17.7%, (13057), [35, 0.3%]	17.7%, (13057), [35, 0.3%]
**NICOLA**	24.0%, (3638), [4642, 56.1%]	24.0%, (3638), [4642, 56.1%]
**KLoSA**	7.1%, (7949), [0, 0.0%]	38.2%, (7949), [0, 0.0%]
**SHARE S**	20.8%, (23793), [57, 0.2%]	20.8%, (23793), [57, 0.2%]
**SHARE E**	23.0%, (6567), [14, 0.2%]	23.0%, (6567), [14, 0.2%]
**MHAS**	33.3%, (14582), [1303, 8.2%]	33.3%, (14582), [1303, 8.2%]
**CHARLS**	14.9%, (17178), [106, 0.6%]	66.2%, (17178), [106, 0.6%]
**MARS**	17.0%, (4053), [4, 0.1%]	17.0%, (4053), [4, 0.1%]
**ELSI**	26.4%, (9807), [142, 1.4%]	26.4%, (9807), [142, 1.4%]
**LASI**	7.4%, (53015), [151, 0.3%]	7.4%, (53015), [151, 0.3%]

High Cholesterol

**High Cholesterol (%, (N), [miss, %miss])**	**Doctor Diagnosed High Cholesterol**
**TILDA**	37.6%, (8163), [12, 0.1%]
**HRS**	53.1%, (20838), [74, 0.4%]
**SHARE W**	19.9%, (21476), [39, 0.2%]
**ELSA**	41.1%, (7474), [8, 0.1%]
**SHARE N**	17.6%, (13055), [37, 0.3%]
**NICOLA**	36.4%, (8201), [79, 1.0%]
**KLoSA**	
**SHARE S**	27.1%, (23802), [48, 0.2%]
**SHARE E**	21.6%, (6565), [16, 0.2%]
**MHAS**	
**CHARLS**	23.0%, (17201), [83, 0.5%]
**MARS**	25.2%, (4053), [4, 0.1%]
**ELSI**	23.0%, (9774), [175, 1.8%]
**LASI**	2.9%, (53027), [139, 0.3%]

Depressive symptoms

Depression (%, (N), [miss, %miss])	Depression
**TILDA**	9.8%, (8171), [4, 0.0%]
**HRS**	19.8%, (19965), [936, 4.6%]
**SHARE W**	28.1%, (21515), [0, 0.0%]
**ELSA**	25.1%, (7017), [465, 6.2%]
**SHARE N**	19.6%, (13092), [0, 0.0%]
**NICOLA**	14.1%, (3492), [4788, 57.8%]
**KLoSA**	23.5%, (7946), [3, 0.0%]
**SHARE S**	32.2%, (23850), [0, 0.0%]
**SHARE E**	37.6%, (6581), [0, 0.0%]
**MHAS**	30.7%, (14589), [1296, 8.2%]
**CHARLS**	37.2%, (15691), [1593, 9.2%]
**MARS**	
**ELSI**	26.0%, (8912), [1037, 10.4%]
**LASI**	12.9%, (51658), [1508, 2.8%]

Physical Inactivity

Physical Inactivty (%, (N), [miss, %miss])	Low Physical Activity (from the Leisure Scale Index)	Low Physical Activity Alt (from the Leisure Scale Index)	No Light Physical Activity	No Moderate Physical Activity	No Vigorous Physical Activity
**TILDA**	24.1%, (8146), [29, 0.4%]	24.1%, (8146), [29, 0.4%]	17.5%, (8161), [14, 0.2%]	52.7%, (8159), [16, 0.2%]	75.3%, (8163), [12, 0.1%]
**HRS**	27.8%, (20710), [199, 1.0%]	30.6%, (20710), [199, 1.0%]	16.1%, (20851), [60, 0.3%]	29.1%, (20830), [82, 0.4%]	61.1%, (20808), [102, 0.5%]
**SHARE W**				17.0%, (21474), [41, 0.2%]	50.8%, (21475), [40, 0.2%]
**ELSA**	24.5%, (7477), [5, 0.1%]	17.0%, (7477), [5, 0.1%]	15.2%, (7479), [3, 0.0%]	26.3%, (7479), [3, 0.0%]	69.4%, (7479), [3, 0.0%]
**SHARE N**				10.5%, (13060), [32, 0.2%]	40.8%, (13059), [33, 0.3%]
**NICOLA**	43.0%, (8152), [128, 1.5%]	43.0%, (8152), [128, 1.5%]	29.8%, (8186), [94, 1.1%]	66.6%, (8173), [107, 1.3%]	80.1%, (8181), [99, 1.2%]
**KLoSA**					65.7%, (7949), [0, 0.0%]
**SHARE S**				29.3%, (23806), [44, 0.2%]	63.3%, (23805), [45, 0.2%]
**SHARE E**				23.9%, (6566), [15, 0.2%]	58.8%, (6567), [14, 0.2%]
**MHAS**					
**CHARLS**	15.4%, (17212), [72, 0.4%]	15.4%, (17212), [72, 0.4%]	17.7%, (17212), [72, 0.4%]	53.2%, (17212), [72, 0.4%]	72.2%, (17212), [72, 0.4%]
**MARS**	31.9%, (4044), [13, 0.3%]	32.8%, (4044), [13, 0.3%]	31.0%, (4044), [13, 0.3%]	50.2%, (4044), [13, 0.3%]	76.6%, (4044), [13, 0.3%]
**ELSI**	52.3%, (9581), [368, 3.7%]	52.3%, (9581), [368, 3.7%]	39.8%, (9719), [230, 2.3%]	62.8%, (9687), [262, 2.6%]	85.4%, (9698), [251, 2.5%]
**LASI**				36.1%, (52729), [437, 0.8%]	65.4%, (52718), [448, 0.8%]

Smoking

Smoking (%, (N), [miss, %miss])	Current or Former Smoker	Never Smoked	Used to Smoke	Currently Smokes
**TILDA**	57.1%, (8174), [1, 0.0%]	42.9%, (8174), [1, 0.0%]	37.7%, (8174), [1, 0.0%]	19.3%, (8174), [1, 0.0%]
**HRS**	53.8%, (20818), [92, 0.5%]	46.2%, (20818), [92, 0.5%]	40.0%, (20818), [92, 0.5%]	13.8%, (20818), [92, 0.5%]
**SHARE W**	58.9%, (2115), [19400, 90.2%]	41.1%, (2115), [19400, 90.2%]	28.5%, (2115), [19400, 90.2%]	30.4%, (2115), [19400, 90.2%]
**ELSA**	53.0%, (6780), [702, 9.4%]	47.0%, (6780), [702, 9.4%]	40.7%, (6780), [702, 9.4%]	12.3%, (6780), [702, 9.4%]
**SHARE N**	54.3%, (1062), [12030, 91.9%]	45.7%, (1062), [12030, 91.9%]	34.8%, (1062), [12030, 91.9%]	19.5%, (1062), [12030, 91.9%]
**NICOLA**	51.6%, (8193), [87, 1.1%]	48.4%, (8193), [87, 1.1%]	35.0%, (8193), [87, 1.1%]	16.6%, (8193), [87, 1.1%]
**KLoSA**	33.4%, (7949), [0, 0.0%]	66.6%, (7949), [0, 0.0%]	17.4%, (7949), [0, 0.0%]	16.0%, (7949), [0, 0.0%]
**SHARE S**	40.5%, (8283), [15567, 65.3%]	59.5%, (8283), [15567, 65.3%]	17.6%, (8283), [15567, 65.3%]	22.9%, (8283), [15567, 65.3%]
**SHARE E**	53.2%, (495), [6086, 92.5%]	46.8%, (495), [6086, 92.5%]	20.6%, (495), [6086, 92.5%]	32.6%, (495), [6086, 92.5%]
**MHAS**	37.3%, (15868), [17, 0.1%]	62.7%, (15868), [17, 0.1%]	24.7%, (15868), [17, 0.1%]	12.7%, (15868), [17, 0.1%]
**CHARLS**	44.0%, (17210), [74, 0.4%]	56.0%, (17210), [74, 0.4%]	17.0%, (17210), [74, 0.4%]	26.9%, (17210), [74, 0.4%]
**MARS**	25.8%, (4053), [4, 0.1%]	74.2%, (4053), [4, 0.1%]	9.0%, (4053), [4, 0.1%]	16.9%, (4053), [4, 0.1%]
**ELSI**	35.7%, (9854), [95, 1.0%]	64.3%, (9854), [95, 1.0%]	21.8%, (9854), [95, 1.0%]	13.8%, (9854), [95, 1.0%]
**LASI**	19.8%, (52719), [447, 0.8%]	80.2%, (52719), [447, 0.8%]	4.6%, (52719), [447, 0.8%]	15.2%, (52719), [447, 0.8%]

Diabetes

**Diabetes (%, (N), [miss, %miss])**	**Doctor Diagnosed with Diabetes**
**TILDA**	8.0%, (8163), [12, 0.1%]
**HRS**	22.6%, (20912), [0, 0.0%]
**SHARE W**	14.5%, (21480), [35, 0.2%]
**ELSA**	12.9%, (7479), [3, 0.0%]
**SHARE N**	11.5%, (13058), [34, 0.3%]
**NICOLA**	11.3%, (8201), [79, 1.0%]
**KLoSA**	15.9%, (7949), [0, 0.0%]
**SHARE S**	17.6%, (23807), [43, 0.2%]
**SHARE E**	16.8%, (6567), [14, 0.2%]
**MHAS**	26.1%, (15862), [23, 0.1%]
**CHARLS**	13.4%, (17205), [79, 0.5%]
**MARS**	23.0%, (4053), [4, 0.1%]
**ELSI**	17.7%, (9842), [107, 1.1%]
**LASI**	13.1%, (53023), [143, 0.3%]

Hypertension

**Hypertension (%, (N), [miss, %miss])**	**Doctor Diagnosed with Hypertension**	**Hypertension using Measured Blood Pressure**
**TILDA**	37.8%, (8163), [12, 0.1%]	63.7%, (6656), [1519, 18.6%]
**HRS**	55.1%, (20912), [0, 0.0%]	75.8%, (14618), [5941, 29.5%]
**SHARE W**	49.5%, (21469), [46, 0.2%]	
**ELSA**	42.4%, (7479), [3, 0.0%]	54.0%, (8654), [1755, 16.9%]
**SHARE N**	46.0%, (13062), [30, 0.2%]	
**NICOLA**	40.6%, (8200), [80, 1.0%]	69.6%, (5357), [2923, 35.3%]
**KLoSA**	36.6%, (7949), [0, 0.0%]	
**SHARE S**	52.8%, (23806), [44, 0.2%]	
**SHARE E**	57.0%, (6568), [13, 0.2%]	
**MHAS**	53.1%, (15871), [13, 0.1%]	78.7%, (6883), [8005, 53.8%]
**CHARLS**	40.2%, (17202), [82, 0.5%]	47.8%, (13599), [2905, 17.6%]
**MARS**	43.4%, (4053), [4, 0.1%]	70.1%, (3977), [80, 2.0%]
**ELSI**	49.0%, (9866), [83, 0.8%]	64.3%, (9176), [773, 7.8%]
**LASI**	28.5%, (53025), [141, 0.3%]	45.4%, (49439), [3727, 7.0%]

High BMI

BMI (%, (N), [miss, %miss])	Obese	BMI Measured or Self Report (mean, (N), [miss, %miss])	BMI Measured (mean, (N), [miss, %miss])	BMI Self Report (mean, (N), [miss, %miss])
**TILDA**	35.3%, (5873), [2300, 28.2%]	28.79, (4.99), [2300, 28.2%]	28.79, (4.99), [2300, 28.2%]	
**HRS**	44.9%, (7749), [12713, 63.1%]	29.99, (6.23), [12713, 63.1%]	29.99, (6.23), [12713, 63.1%]	28.82, (6.20), [307, 1.5%]
**SHARE W**	21.3%, (21115), [400, 1.9%]	26.77, (4.84), [400, 1.9%]		26.77, (4.84), [400, 1.9%]
**ELSA**	32.2%, (7663), [2709, 26.1%]	28.40, (5.32), [2709, 26.1%]	28.40, (5.32), [2709, 26.1%]	
**SHARE N**	18.2%, (12832), [260, 2.0%]	26.37, (4.37), [260, 2.0%]		26.37, (4.37), [260, 2.0%]
**NICOLA**	35.5%, (3630), [4650, 56.2%]	28.96, (5.21), [4650, 56.2%]	28.96, (5.21), [4650, 56.2%]	
**KLoSA**	6.2%, (7814), [135, 1.7%]	23.32, (2.68), [135, 1.7%]		23.32, (2.68), [135, 1.7%]
**SHARE S**	18.2%, (23040), [810, 3.4%]	26.58, (4.28), [810, 3.4%]		26.58, (4.28), [810, 3.4%]
**SHARE E**	29.4%, (6411), [170, 2.6%]	27.70, (4.97), [170, 2.6%]		27.70, (4.97), [170, 2.6%]
**MHAS**	35.7%, (1893), [12993, 87.3%]	28.43, (5.13), [12993, 87.3%]	28.43, (5.13), [12993, 87.3%]	27.74, (5.18), [1795, 11.3%]
**CHARLS**	14.5%, (12973), [3531, 21.4%]	23.77, (3.91), [3531, 21.4%]	23.77, (3.91), [3531, 21.4%]	
**MARS**	36.1%, (3771), [286, 7.0%]	26.53, (5.16), [286, 7.0%]	26.53, (5.16), [286, 7.0%]	
**ELSI**	30.7%, (8420), [1529, 15.4%]	27.93, (5.26), [1529, 15.4%]	27.93, (5.26), [1529, 15.4%]	
**LASI**	13.3%, (47715), [5451, 10.3%]	22.34, (4.72), [5451, 10.3%]	22.34, (4.72), [5451, 10.3%]	

Alcohol Consumption

Alcohol (%, (N), [miss, %miss])	Excessive Drinking	Binge Drinking
**TILDA**	13.6%, (6394), [1774, 21.7%]	22.5%, (6521), [1647, 20.2%]
**HRS**	8.0%, (20784), [123, 0.6%]	4.1%, (20828), [81, 0.4%]
**SHARE W**	12.4%, (21456), [59, 0.3%]	5.1%, (28274), [142, 0.5%]
**ELSA**	16.8%, (6310), [1172, 15.7%]	18.8%, (7361), [1977, 21.2%]
**SHARE N**	10.5%, (13038), [54, 0.4%]	7.2%, (14118), [130, 0.9%]
**NICOLA**		
**KLoSA**	13.8%, (7902), [47, 0.6%]	24.1%, (7949), [0, 0.0%]
**SHARE S**	7.9%, (23791), [59, 0.2%]	3.0%, (14057), [64, 0.5%]
**SHARE E**	5.4%, (6559), [22, 0.3%]	14.7%, (5477), [40, 0.7%]
**MHAS**	3.7%, (15736), [149, 0.9%]	6.1%, (15806), [79, 0.5%]
**CHARLS**		
**MARS**	0.6%, (4050), [7, 0.2%]	1.4%, (4041), [16, 0.4%]
**ELSI**	4.1%, (9753), [196, 2.0%]	6.1%, (9758), [191, 1.9%]
**LASI**		5.1%, (52720), [446, 0.8%]

Social Isolation

**Social Isolation (%, (N), [miss, %miss])**	**Living alone**
**TILDA**	22.9%, (8163), [12, 0.1%]
**HRS**	22.3%, (20912), [0, 0.0%]
**SHARE W**	29.7%, (21515), [0, 0.0%]
**ELSA**	22.6%, (7482), [0, 0.0%]
**SHARE N**	33.4%, (13092), [0, 0.0%]
**NICOLA**	26.1%, (8280), [0, 0.0%]
**KLoSA**	13.1%, (7949), [0, 0.0%]
**SHARE S**	23.2%, (23850), [0, 0.0%]
**SHARE E**	23.2%, (6581), [0, 0.0%]
**MHAS**	10.8%, (15885), [0, 0.0%]
**CHARLS**	10.0%, (17284), [0, 0.0%]
**MARS**	4.9%, (4057), [0, 0.0%]
**ELSI**	21.2%, (9949), [0, 0.0%]
**LASI**	3.9%, (53166), [0, 0.0%]

Poor Vision

Vision (%, (N), [miss, %miss])	Poor Vision Base Scale	Poor Vision Alt Scale
**TILDA**	11.0%, (8175), [0, 0.0%]	11.0%, (8175), [0, 0.0%]
**HRS**	21.0%, (20794), [113, 0.6%]	21.0%, (20794), [113, 0.6%]
**SHARE W**	20.2%, (21450), [65, 0.3%]	20.2%, (21450), [65, 0.3%]
**ELSA**	15.7%, (7478), [4, 0.1%]	15.7%, (7478), [4, 0.1%]
**SHARE N**	16.4%, (13049), [43, 0.3%]	16.4%, (13049), [43, 0.3%]
**NICOLA**	15.4%, (3638), [4642, 56.1%]	15.4%, (3638), [4642, 56.1%]
**KLoSA**	17.8%, (7949), [0, 0.0%]	72.9%, (7949), [0, 0.0%]
**SHARE S**	31.4%, (23789), [61, 0.3%]	31.4%, (23789), [61, 0.3%]
**SHARE E**	38.8%, (6561), [20, 0.3%]	38.8%, (6561), [20, 0.3%]
**MHAS**	47.2%, (14565), [1320, 8.3%]	47.2%, (14565), [1320, 8.3%]
**CHARLS**	30.4%, (17161), [123, 0.7%]	82.3%, (17178), [106, 0.6%]
**MARS**	30.8%, (4053), [4, 0.1%]	30.8%, (4053), [4, 0.1%]
**ELSI**	49.6%, (9864), [85, 0.9%]	49.6%, (9864), [85, 0.9%]
**LASI**	69.7%, (52969), [197, 0.4%]	69.7%, (52969), [197, 0.4%]

Appendix 6: Confidence intervals for prevalence estimates

Table S6.1. Weighted prevalence of 12 risk factors for dementia across 14 geographical countries and regions

	TILDA	HRS	SHARE W	ELSA	SHARE N	NICOLA	KLoSA	SHARE S	SHARE E	MHAS	CHARLS	MARS	ELSI	LASI
	Ireland	USA	Western Europe	England	Northern Europe	Northern Ireland	South Korea	Southern Europe	Eastern Europe	Mexico	China	Malaysia	Brazil	India
Low education	63.4% (95% CI 61.9, 64.9) (8171)	12.0% (95% CI 11.3, 12.7) (20901)	24.6% (95% CI 18.9, 30.4) (21515)	20.4% (95% CI 19.0, 21.8) (6864)	26.4% (95% CI 18.8, 34.1) (13092)	62.5% (95% CI 61.5, 63.6) (8214)	47.7% (95% CI 46.4, 48.9) (7949)	69.5% (95% CI 67.5, 71.5) (23850)	34.0% (95% CI 26.1, 41.9) (6581)	84.0% (95% CI 82.7, 85.3) (15651)	85.6% (95% CI 84.8, 86.5) (17284)	70.0% (95% CI 68.5, 71.4) (4057)	76.4% (95% CI 75.2, 77.6) (9781)	76.7% (95% CI 76.3, 77.2) (53166)
Hearing loss	15.2% (95% CI 14.2, 16.1) (8175)	19.4% (95% CI 19.2, 19.7) (20885)	20.4% (95% CI 19.0, 21.7) (21469)	21.0% (95% CI 19.7, 22.2) (7480)	17.7% (95% CI 14.9, 20.5) (13057)	24.0% (95% CI 22.6, 25.4) (3638)	7.1% (95% CI 6.6, 7.7) (7949)	20.8% (95% CI 19.5, 22.1) (23793)	23.0% (95% CI 17.3, 28.8) (6567)	33.3% (95% CI 31.7, 34.8) (14582)	14.9% (95% CI 14.2, 15.5) (17178)	17.0% (95% CI 15.8, 18.1) (4053)	26.4% (95% CI 25.3, 27.5) (9807)	7.4% (95% CI 7.1, 7.6) (53015)
High LDL cholesterol	37.6% (95% CI 36.2, 38.9) (8163)	53.1% (95% CI 51.2, 55.0) (20838)	19.9% (95% CI 18.9, 20.9) (21476)	41.1% (95% CI 39.6, 42.7) (7474)	17.6% (95% CI 16.9, 18.3) (13055)	36.4% (95% CI 35.4, 37.5) (8201)		27.1% (95% CI 25.8, 28.4) (23802)	21.6% (95% CI 18.9, 24.4) (6565)		23.0% (95% CI 22.1, 23.9) (17201)	25.2% (95% CI 23.8, 26.5) (4053)	23.0% (95% CI 21.9, 24.1) (9774)	2.9% (95% CI 2.7, 3.0) (53027)
Depression	9.8% (95% CI 9.0, 10.6) (8171)	19.8% (95% CI 19.1, 20.5) (19965)	28.1% (95% CI 26.1, 30.2) (21515)	25.1% (95% CI 23.6, 26.6) (7017)	19.6% (95% CI 17.1, 22.2) (13092)	14.1% (95% CI 12.9, 15.2) (3492)	23.5% (95% CI 22.4, 24.6) (7946)	32.2% (95% CI 31.0, 33.5) (23850)	37.6% (95% CI 32.3, 42.9) (6581)	30.7% (95% CI 29.1, 32.4) (14589)	37.2% (95% CI 36.2, 38.3) (15691)		26.0% (95% CI 24.8, 27.3) (8912)	12.9% (95% CI 12.6, 13.3) (51658)
Physical inactivity		27.8% (95% CI 27.7, 28.0) (20710)		24.5% (95% CI 23.1, 25.9) (7477)		43.0% (95% CI 41.9, 44.1) (8152)					15.4% (95% CI 14.7, 16.2) (17212)	31.9% (95% CI 30.5, 33.4) (4044)	52.3% (95% CI 51.0, 53.7) (9581)	
Smoking	57.1% (95% CI 55.7, 58.4) (8174)	53.8% (95% CI 52.3, 55.3) (20818)	58.9% (95% CI 54.8, 62.9) (2115)	53.0% (95% CI 51.3, 54.7) (6780)	54.3% (95% CI 52.6, 56.0) (1062)	51.6% (95% CI 50.5, 52.7) (8193)	33.4% (95% CI 32.1, 34.7) (7949)	40.5% (95% CI 38.2, 42.8) (8283)	53.2% (95% CI 47.2, 59.2) (495)	37.3% (95% CI 35.7, 39.0) (15868)	44.0% (95% CI 42.9, 45.0) (17210)	25.8% (95% CI 24.5, 27.2) (4053)	35.7% (95% CI 34.4, 37.0) (9854)	19.8% (95% CI 19.4, 20.3) (52719)
Diabetes	8.0% (95% CI	22.6% (95% CI	14.5% (95% CI	12.9% (95% CI	11.5% (95% CI	11.3% (95% CI	15.9% (95% CI	17.6% (95% CI	16.8% (95% CI	26.1% (95% CI	13.4% (95% CI	23.0% (95% CI	17.7% (95% CI	13.1% (95% CI

	7.4, 8.6) (8163)	22.3, 23.0) (20912)	13.8, 15.2) (21480)	11.8, 13.9) (7479)	8.9, 14.1) (13058)	10.6, 12.0) (8201)	15.0, 16.8) (7949)	16.5, 18.8) (23807)	14.1, 19.5) (6567)	24.7, 27.4) (15862)	12.6, 14.1) (17205)	21.7, 24.3) (4053)	16.6, 18.7) (9842)	12.8, 13.5) (53023)
Hypertension	37.8% (95% CI 36.6, 38.9) (8163)	55.0% (95% CI 54.6, 55.4) (20912)	46.7% (95% CI 44.5, 48.8) (21487)	41.8% (95% CI 40.3, 43.4) (7481)	44.1% (95% CI 38.7, 49.4) (13073)	40.6% (95% CI 39.5, 41.6) (8201)	36.6% (95% CI 35.4, 37.8) (7949)	50.1% (95% CI 48.4, 51.9) (23809)	54.3% (95% CI 49.2, 59.3) (6571)	53.1% (95% CI 51.5, 54.7) (15871)	40.2% (95% CI 39.1, 41.2) (17202)	43.4% (95% CI 41.8, 44.9) (4053)	49.0% (95% CI 47.7, 50.3) (9866)	28.5% (95% CI 28.0, 29.0) (53025)
Obesity	35.3% (95% CI 34.0, 36.6) (5873)	44.9% (95% CI 43.3, 46.5) (7749)	21.3% (95% CI 20.3, 22.3) (21115)	32.2% (95% CI 30.9, 33.5) (7663)	18.2% (95% CI 16.7, 19.8) (12832)	35.5% (95% CI 34.0, 37.1) (3630)	6.2% (95% CI 5.6, 6.8) (7814)	18.2% (95% CI 17.0, 19.4) (23040)	29.4% (95% CI 27.1, 31.7) (6411)	35.7% (95% CI 31.5, 39.9) (1893)	14.5% (95% CI 13.7, 15.3) (12973)	36.1% (95% CI 34.6, 37.7) (3771)	30.7% (95% CI 29.4, 32.1) (8420)	13.3% (95% CI 12.9, 13.7) (47715)
Alcohol consumption	13.6% (95% CI 12.6, 14.6) (6394)	8.0% (95% CI 6.9, 9.1) (20784)	12.4% (95% CI 11.4, 13.4) (21456)	16.8% (95% CI 15.6, 18.1) (6310)	10.5% (95% CI 7.3, 13.8) (13038)		13.8% (95% CI 12.8, 14.8) (7902)	7.9% (95% CI 7.0, 8.7) (23791)	5.4% (95% CI 3.7, 7.1) (6559)	3.7% (95% CI 3.1, 4.3) (15736)		0.6% (95% CI 0.4, 0.9) (4050)	4.1% (95% CI 3.5, 4.7) (9753)	
Social isolation	22.9% (95% CI 21.8, 24.0) (8163)	22.3% (95% CI 20.5, 24.0) (20912)	29.7% (95% CI 28.0, 31.4) (21515)	22.6% (95% CI 21.3, 23.9) (7482)	33.4% (95% CI 28.1, 38.6) (13092)	26.1% (95% CI 25.2, 27.1) (8280)	13.1% (95% CI 12.3, 14.0) (7949)	23.2% (95% CI 21.9, 24.5) (23850)	23.2% (95% CI 19.2, 27.3) (6581)	10.8% (95% CI 9.5, 12.1) (15885)	10.0% (95% CI 9.3, 10.6) (17284)	4.9% (95% CI 4.2, 5.5) (4057)	21.2% (95% CI 20.3, 22.1) (9949)	3.9% (95% CI 3.7, 4.1) (53166)
Vision loss	11.0% (95% CI 10.2, 11.9) (8175)	21.0% (95% CI 20.7, 21.4) (20794)	20.2% (95% CI 16.5, 23.9) (21450)	15.7% (95% CI 14.5, 16.9) (7478)	16.4% (95% CI 12.4, 20.4) (13049)	15.4% (95% CI 14.2, 16.5) (3638)	17.8% (95% CI 16.9, 18.7) (7949)	31.4% (95% CI 29.8, 32.9) (23789)	38.8% (95% CI 32.9, 44.6) (6561)	47.2% (95% CI 45.5, 48.9) (14565)	30.4% (95% CI 29.5, 31.3) (17161)	30.8% (95% CI 29.4, 32.3) (4053)	49.6% (95% CI 48.3, 50.9) (9864)	69.7% (95% CI 69.2, 70.2) (52969)

All data are in percentages. The regions are ordered by per capita gross domestic product (high->low) from left to right. CHARLS=China Health and Retirement Longitudinal Study. E=East. ELSA=English Longitudinal Study of Ageing. ELSI=Brazilian Longitudinal Study of Ageing. HRS=Health and Retirement Study. KLoSA=Korean Longitudinal Study of Ageing. LASI=Longitudinal Ageing Study in India. MARS=Malaysia Ageing and Retirement Survey. MHAS=Mexican Health and Aging Study. N=North. NICOLA=Northern Ireland Cohort for the Longitudinal Study of Ageing. S=South. SHARE=Survey of Health, Ageing and Retirement in Europe. TILDA=The Irish Longitudinal Study on Ageing. W=West. *Cross-national challenges identify potential methodological or measurement reasons behind observed differences across countries or regions.

Appendix 7: Patterns in risk factor prevalence by demographic variables

The following tables contain data displayed in Figure 2.

Age

Risk Factor	TILDA (Ireland)	HRS (US)	SHARE W. (W Europe)	ELSA (England)	SHARE N. (N Europe)	NICOLA (N Ireland)	KLoSA (South Korea)	SHARE S. (S Europe)	SHARE E. (E Europe)	MHAS (Mexico)	CHARLS (China)	MARS (Malaysia)	ELSI (Brazil)	LASI (India)
Less than Secondary Education	1.37 (1.32, 1.41)	1.82 (1.55, 2.14)	2.02 (1.84, 2.21)	2.57 (2.22, 2.98)	2.60 (2.20, 3.07)	1.25 (1.21, 1.29)	2.13 (2.04, 2.23)	1.40 (1.35, 1.46)	2.47 (2.16, 2.83)	1.15 (1.12, 1.18)	1.06 (1.04, 1.08)	1.30 (1.25, 1.35)	1.19 (1.16, 1.22)	1.13 (1.12, 1.15)
Poor Hearing	1.84 (1.65, 2.06)	1.70 (1.59, 1.82)	2.06 (1.88, 2.25)	1.99 (1.76, 2.26)	1.85 (1.64, 2.07)	1.26 (1.12, 1.42)	4.62 (3.73, 5.73)	2.73 (2.43, 3.07)	2.63 (2.16, 3.20)	1.48 (1.36, 1.61)	2.56 (2.34, 2.80)	2.44 (2.13, 2.79)	1.58 (1.46, 1.71)	2.37 (2.20, 2.55)
High Cholesterol	0.98 (0.92, 1.05)	1.17 (1.13, 1.22)	1.34 (1.23, 1.46)	1.70 (1.57, 1.83)	1.40 (1.15, 1.70)	1.17 (1.11, 1.24)		1.25 (1.15, 1.35)	1.33 (1.08, 1.64)		1.02 (0.94, 1.11)	1.10 (0.96, 1.25)	1.02 (0.92, 1.12)	1.02 (0.89, 1.18)
Depression	0.83 (0.70, 0.97)	0.85 (0.79, 0.92)	1.06 (1.00, 1.13)	0.82 (0.73, 0.92)	1.09 (0.93, 1.28)	0.85 (0.70, 1.04)	1.57 (1.43, 1.72)	1.42 (1.32, 1.52)	1.36 (1.21, 1.52)	1.20 (1.09, 1.32)	1.12 (1.05, 1.19)		0.92 (0.83, 1.01)	1.27 (1.19, 1.35)
Low Physical Activity (met. minutes)		1.65 (1.52, 1.79)		1.45 (1.29, 1.63)		1.29 (1.22, 1.35)					2.14 (1.95, 2.35)	1.60 (1.46, 1.76)	1.33 (1.27, 1.39)	
Smoking (Former or Current)	0.93 (0.88, 0.97)	1.05 (1.00, 1.09)	0.83 (0.69, 0.98)	1.37 (1.29, 1.46)		0.87 (0.83, 0.91)	0.88 (0.83, 0.93)	0.59 (0.52, 0.68)	0.63 (0.31, 1.27)	0.90 (0.84, 0.98)	1.04 (1.00, 1.08)	0.94 (0.84, 1.05)	0.94 (0.87, 1.01)	0.98 (0.93, 1.02)
Diabetes	1.64 (1.40, 1.93)	1.30 (1.22, 1.38)	1.71 (1.50, 1.94)	1.26 (1.07, 1.50)	1.55 (1.32, 1.81)	1.48 (1.31, 1.68)	1.67 (1.47, 1.88)	1.92 (1.72, 2.14)	1.92 (1.56, 2.35)	1.13 (1.02, 1.25)	1.11 (0.98, 1.25)	1.17 (1.02, 1.34)	1.27 (1.14, 1.42)	1.21 (1.14, 1.29)
Hypertension (Doctor Diagnosed)	1.53 (1.45, 1.63)	1.43 (1.37, 1.49)	1.59 (1.52, 1.66)	1.63 (1.51, 1.76)	1.61 (1.49, 1.74)	1.44 (1.37, 1.52)	1.73 (1.62, 1.85)	1.70 (1.61, 1.78)	1.54 (1.41, 1.67)	1.50 (1.42, 1.58)	1.47 (1.40, 1.54)	1.36 (1.26, 1.47)	1.32 (1.26, 1.39)	1.39 (1.34, 1.43)
Obese	0.97 (0.89, 1.06)	0.78 (0.73, 0.84)	0.83 (0.76, 0.91)	0.77 (0.68, 0.89)	0.74 (0.64, 0.86)	0.87 (0.79, 0.97)	0.84 (0.68, 1.04)	0.92 (0.83, 1.02)	0.90 (0.80, 1.01)	0.88 (0.60, 1.30)	0.69 (0.57, 0.83)	0.66 (0.57, 0.75)	0.71 (0.65, 0.78)	0.62 (0.57, 0.67)
Excessive Drinking	0.50 (0.42, 0.61)	0.56 (0.48, 0.64)	0.88 (0.77, 1.01)	0.74 (0.63, 0.86)	0.80 (0.61, 1.05)		0.48 (0.40, 0.57)	0.79 (0.66, 0.94)	0.70 (0.47, 1.04)	0.35 (0.23, 0.54)		0.58 (0.17, 1.97)	0.36 (0.25, 0.52)	
Lives Alone	2.41 (2.20, 2.64)	1.64 (1.53, 1.77)	1.82 (1.68, 1.97)	1.56 (1.39, 1.76)	1.68 (1.49, 1.88)	1.95 (1.82, 2.10)	2.32 (2.01, 2.67)	2.18 (1.96, 2.43)	1.89 (1.59, 2.25)	1.73 (1.36, 2.18)	3.53 (3.08, 4.05)	2.46 (1.83, 3.30)	1.39 (1.28, 1.52)	2.21 (1.99, 2.45)
Poor Vision	1.52 (1.32, 1.76)	1.07 (0.99, 1.16)	1.32 (1.19, 1.46)	1.26 (1.08, 1.47)	1.61 (1.38, 1.88)	1.06 (0.90, 1.25)	2.50 (2.24, 2.79)	1.56 (1.42, 1.71)	1.43 (1.22, 1.68)	1.16 (1.09, 1.24)	1.32 (1.24, 1.40)	1.37 (1.23, 1.51)	1.04 (0.99, 1.10)	1.16 (1.15, 1.18)

Gender

Risk Factor	TILDA (Ireland)	HRS (US)	SHARE W. (W Europe)	ELSA (England)	SHARE N. (N Europe)	NICOLA (N Ireland)	KLoSA (South Korea)	SHARE S. (S Europe)	SHARE E. (E Europe)	MHAS (Mexico)	CHARLS (China)	MARS (Malaysia)	ELSI (Brazil)	LASI (India)
Less than Secondary Education	0.97 (0.94, 1.00)	0.99 (0.91, 1.08)	1.59 (1.32, 1.93)	1.27 (1.11, 1.45)	0.99 (0.85, 1.15)	0.98 (0.95, 1.02)	1.62 (1.53, 1.71)	1.05 (1.02, 1.09)	1.23 (1.08, 1.40)	1.11 (1.08, 1.15)	1.10 (1.08, 1.12)	1.16 (1.12, 1.21)	0.99 (0.96, 1.02)	1.33 (1.31, 1.34)
Poor Hearing	0.62 (0.56, 0.68)	0.64 (0.60, 0.69)	0.75 (0.70, 0.81)	0.74 (0.65, 0.83)	0.69 (0.61, 0.79)	0.62 (0.55, 0.70)	0.74 (0.62, 0.87)	0.83 (0.76, 0.89)	0.78 (0.64, 0.95)	0.89 (0.82, 0.98)	1.03 (0.94, 1.12)	1.02 (0.89, 1.17)	0.88 (0.81, 0.96)	0.96 (0.89, 1.04)
High Cholesterol	1.11 (1.04, 1.17)	0.98 (0.95, 1.01)	1.00 (0.92, 1.08)	0.93 (0.87, 1.00)	0.98 (0.87, 1.11)	0.93 (0.88, 0.99)		1.09 (1.00, 1.18)	1.45 (1.23, 1.71)		1.15 (1.06, 1.24)	1.23 (1.11, 1.38)	1.58 (1.42, 1.76)	1.51 (1.34, 1.70)
Depression	1.77 (1.53, 2.04)	1.28 (1.19, 1.38)	1.65 (1.55, 1.76)	1.32 (1.17, 1.50)	1.80 (1.64, 1.98)	1.40 (1.19, 1.66)	1.10 (1.00, 1.22)	1.91 (1.77, 2.06)	1.40 (1.27, 1.55)	1.65 (1.45, 1.87)	1.42 (1.34, 1.51)		1.57 (1.42, 1.75)	1.22 (1.15, 1.30)
Low Physical Activity (met. minutes)		1.08 (1.03, 1.13)		1.02 (0.92, 1.14)		1.28 (1.22, 1.35)					1.11 (1.01, 1.22)	0.82 (0.75, 0.89)	1.06 (1.00, 1.11)	
Smoking (Former or Current)	0.78 (0.75, 0.81)	0.83 (0.80, 0.86)	0.83 (0.71, 0.97)	0.86 (0.81, 0.92)		0.74 (0.71, 0.78)	0.06 (0.05, 0.07)	0.55 (0.49, 0.62)	0.70 (0.54, 0.90)	0.37 (0.33, 0.41)	0.10 (0.09, 0.11)	0.04 (0.03, 0.05)	0.62 (0.57, 0.67)	0.11 (0.10, 0.11)
Diabetes	0.65 (0.56, 0.76)	0.88 (0.83, 0.94)	0.69 (0.63, 0.76)	0.82 (0.70, 0.97)	0.77 (0.62, 0.94)	0.68 (0.60, 0.77)	0.82 (0.73, 0.92)	0.81 (0.74, 0.89)	0.96 (0.75, 1.23)	1.22 (1.09, 1.36)	1.16 (1.03, 1.29)	1.04 (0.92, 1.16)	1.21 (1.07, 1.36)	1.17 (1.10, 1.23)
Hypertension (Doctor Diagnosed)	1.05 (0.99, 1.11)	0.92 (0.89, 0.95)	0.95 (0.91, 0.98)	0.84 (0.78, 0.90)	0.98 (0.91, 1.05)	0.95 (0.90, 1.00)	1.02 (0.95, 1.10)	0.98 (0.93, 1.03)	1.15 (1.07, 1.24)	1.32 (1.24, 1.41)	1.08 (1.03, 1.14)	1.25 (1.16, 1.34)	1.25 (1.18, 1.32)	1.52 (1.47, 1.57)
Obese	0.86 (0.80, 0.93)	1.04 (0.98, 1.11)	0.93 (0.86, 1.00)	1.17 (1.04, 1.32)	1.06 (0.93, 1.22)	0.90 (0.83, 0.98)	1.28 (1.03, 1.58)	1.16 (1.05, 1.29)	1.06 (0.94, 1.20)	1.17 (0.89, 1.54)	1.42 (1.26, 1.60)	1.42 (1.30, 1.56)	1.42 (1.29, 1.57)	2.51 (2.36, 2.67)
Excessive Drinking	0.90 (0.80, 1.01)	0.96 (0.84, 1.09)	0.59 (0.54, 0.66)	1.22 (1.05, 1.43)	1.06 (0.93, 1.21)		0.16 (0.13, 0.19)	0.44 (0.37, 0.52)	0.27 (0.18, 0.40)	0.18 (0.11, 0.27)		0.15 (0.05, 0.45)	0.34 (0.24, 0.48)	
Lives Alone	1.15 (1.07, 1.24)	1.33 (1.22, 1.45)	1.40 (1.27, 1.53)	1.22 (1.09, 1.37)	1.55 (1.34, 1.80)	1.34 (1.25, 1.45)	2.10 (1.77, 2.50)	1.72 (1.54, 1.93)	1.56 (1.11, 2.19)	1.22 (0.93, 1.60)	1.35 (1.17, 1.55)	1.59 (1.19, 2.13)	1.18 (1.08, 1.30)	2.97 (2.62, 3.38)
Poor Vision	1.06 (0.92, 1.21)	1.06 (0.99, 1.15)	1.36 (1.17, 1.56)	1.13 (0.97, 1.33)	1.03 (0.81, 1.30)	0.89 (0.77, 1.04)	1.20 (1.07, 1.33)	1.17 (1.09, 1.25)	1.00 (0.90, 1.12)	0.93 (0.87, 1.00)	1.25 (1.18, 1.33)	1.04 (0.95, 1.14)	0.99 (0.94, 1.05)	1.06 (1.05, 1.08)

Education

Risk Factor	TILDA (Ireland)	HRS (US)	SHARE W. (W Europe)	ELSA (England)	SHARE N. (N Europe)	NICOLA (N Ireland)	KLoSA (South Korea)	SHARE S. (S Europe)	SHARE E. (E Europe)	MHAS (Mexico)	CHARLS (China)	MARS (Malaysia)	ELSI (Brazil)	LASI (India)
Less than Secondary Education	1.46 (1.30, 1.65)	1.78 (1.63, 1.95)	1.12 (1.03, 1.22)	1.17 (1.02, 1.35)	1.20 (1.11, 1.29)	1.26 (1.12, 1.41)	1.47 (1.16, 1.86)	1.39 (1.19, 1.63)	1.22 (0.97, 1.53)	1.77 (1.49, 2.11)	1.77 (1.47, 2.14)	1.33 (1.11, 1.59)	1.39 (1.22, 1.58)	0.98 (0.89, 1.07)
Poor Hearing	0.93 (0.87, 0.98)	1.00 (0.95, 1.05)	1.19 (1.09, 1.31)	1.14 (1.04, 1.24)	1.03 (0.96, 1.10)	1.10 (1.03, 1.17)		1.20 (1.08, 1.35)	1.03 (0.89, 1.18)		0.63 (0.56, 0.70)	1.10 (0.97, 1.25)	1.03 (0.91, 1.16)	0.29 (0.26, 0.33)
High Cholesterol	1.52 (1.30, 1.77)	1.95 (1.79, 2.14)	1.46 (1.36, 1.55)	1.67 (1.45, 1.92)	1.16 (1.02, 1.31)	1.51 (1.27, 1.79)	1.27 (1.13, 1.43)	1.40 (1.27, 1.55)	1.31 (1.15, 1.49)	1.44 (1.13, 1.83)	1.63 (1.43, 1.85)		1.38 (1.21, 1.57)	1.45 (1.34, 1.57)
Depression		1.67 (1.58, 1.76)		2.00 (1.77, 2.26)		1.42 (1.34, 1.51)					1.33 (1.12, 1.58)	0.98 (0.88, 1.09)	1.17 (1.08, 1.26)	
Low Physical Activity (met. minutes)	1.11 (1.07, 1.16)	1.18 (1.13, 1.23)	1.07 (0.91, 1.24)	1.22 (1.13, 1.31)		1.23 (1.17, 1.29)	1.18 (1.12, 1.25)	0.96 (0.86, 1.08)	1.06 (0.77, 1.46)	0.93 (0.83, 1.04)	1.06 (1.01, 1.10)	1.14 (1.04, 1.25)	1.12 (1.01, 1.24)	1.73 (1.65, 1.82)
Smoking (Former or Current)	1.29 (1.09, 1.53)	1.51 (1.37, 1.66)	1.28 (1.14, 1.43)	1.51 (1.23, 1.85)	1.21 (1.12, 1.31)	1.50 (1.30, 1.72)	1.33 (1.15, 1.54)	1.64 (1.42, 1.90)	1.34 (1.05, 1.70)	1.03 (0.85, 1.24)	0.74 (0.64, 0.85)	0.93 (0.82, 1.06)	1.10 (0.92, 1.30)	0.46 (0.44, 0.49)
Diabetes	1.11 (1.04, 1.18)	1.19 (1.15, 1.23)	1.05 (1.00, 1.10)	1.19 (1.09, 1.29)	1.10 (1.05, 1.14)	1.17 (1.11, 1.24)	1.16 (1.07, 1.26)	1.26 (1.18, 1.34)	1.08 (1.00, 1.18)	1.13 (1.00, 1.26)	0.94 (0.87, 1.03)	1.10 (1.01, 1.20)	1.17 (1.09, 1.27)	0.64 (0.62, 0.67)
Hypertension (Doctor Diagnosed)	1.24 (1.15, 1.34)	1.01 (0.92, 1.11)	1.39 (1.28, 1.51)	1.41 (1.24, 1.60)	1.31 (1.08, 1.58)	1.25 (1.15, 1.37)	1.62 (1.28, 2.05)	1.74 (1.51, 2.02)	1.12 (0.98, 1.28)	1.24 (0.80, 1.93)	0.98 (0.83, 1.16)	0.95 (0.86, 1.04)	0.97 (0.87, 1.08)	0.39 (0.36, 0.41)
Obese	0.75 (0.65, 0.86)	0.89 (0.73, 1.08)	0.77 (0.67, 0.89)	0.61 (0.47, 0.80)	0.54 (0.46, 0.63)		1.11 (0.95, 1.29)	1.19 (0.96, 1.46)	0.94 (0.62, 1.41)	1.17 (0.75, 1.83)		0.77 (0.32, 1.81)	0.63 (0.46, 0.86)	
Excessive Drinking	1.03 (0.94, 1.12)	0.93 (0.85, 1.03)	1.05 (0.94, 1.17)	1.23 (1.07, 1.41)	1.19 (1.08, 1.32)	1.20 (1.11, 1.30)	1.19 (0.99, 1.42)	0.79 (0.69, 0.89)	0.92 (0.74, 1.14)	0.92 (0.69, 1.23)	1.02 (0.80, 1.29)	0.69 (0.50, 0.95)	1.03 (0.92, 1.16)	1.28 (1.10, 1.49)
Lives Alone	1.97 (1.70, 2.30)	2.76 (2.57, 2.95)	1.46 (1.34, 1.58)	1.87 (1.57, 2.23)	1.36 (1.12, 1.66)	1.53 (1.30, 1.80)	1.38 (1.21, 1.58)	1.37 (1.22, 1.54)	1.22 (1.10, 1.36)	1.82 (1.55, 2.15)	1.54 (1.34, 1.78)	1.48 (1.31, 1.68)	1.32 (1.21, 1.43)	1.16 (1.14, 1.19)
Poor Vision	1.46 (1.30, 1.65)	1.78 (1.63, 1.95)	1.12 (1.03, 1.22)	1.17 (1.02, 1.35)	1.20 (1.11, 1.29)	1.26 (1.12, 1.41)	1.47 (1.16, 1.86)	1.39 (1.19, 1.63)	1.22 (0.97, 1.53)	1.77 (1.49, 2.11)	1.77 (1.47, 2.14)	1.33 (1.11, 1.59)	1.39 (1.22, 1.58)	0.98 (0.89, 1.07)

Appendix 8: Gender-education interactions

	Less than Secondary Education	Poor Hearing	High Cholesterol	Depression	Low Physical Activity (met. minutes)	Smoking (Former or Current)	Diabetes	Hypertension (Doctor Diagnosed)	Obese	Excessive Drinking	Lives Alone	Poor Vision
TILDA (Ireland)		1.20	1.35	1.24		1.02	1.83	1.31	1.16	0.99	0.77	1.23
HRS (US)		1.24	1.07	1.08	0.87	0.78	1.39	1.21	1.04	0.40	1.13	1.05
SHARE W. (W Europe)		0.96	1.17	0.95		0.99	1.24	1.27	1.24	0.74	1.01	0.82
ELSA (England)		1.04	1.55	0.90	0.94	0.85	1.29	1.25	0.85	0.55	0.72	0.89
SHARE N. (N Europe)		1.14	1.24	0.91		1.01	1.16	1.03	1.11	1.22	0.90	1.15
NICOLA (N Ireland)		1.33	1.29	0.88	1.04	0.98	1.52	1.24	1.04		0.80	0.78
KLoSA (Korea)		0.93		0.95		1.77	1.60	1.50	2.08	1.15	1.60	1.05
SHARE S. (S Europe)		1.21	1.16	1.02		0.58	1.24	1.15	1.17	0.62	0.86	1.09
SHARE E. (E Europe)		1.11	1.03	1.10		0.79	1.29	1.13	1.36	0.70	0.96	1.14
MHAS (Mexico)		1.37		1.11		0.50	1.43	1.23	0.90	1.13	0.96	0.98
CHARLS (China)		0.90	0.95	1.28	1.20	2.43	1.55	1.02	1.59		1.00	1.23
MARS (Malaysia)		1.12	1.17		1.12	2.27	1.55	1.27	1.31	1.61	0.97	1.14
ELSI (Brazil)		1.03	1.05	0.78	1.10	0.89	1.43	1.21	1.19	1.39	1.05	0.83
LASI (India)		0.84	0.81	1.08		5.98	1.01	1.12	1.13		1.14	1.02

Ratio of RR: 3+, 2, 1, 0

Figure S7.1. Magnitude of education-gender interaction across geographies. The ratio of risk ratios (RRR) shows how much larger or smaller the education gradient is for women compared to men. An RRR greater than 1 indicates that the risk ratio for low education is larger for women than men. The cell border is present for interaction terms that reached statistical significance (p<0.05).
